# Prosaposin is a regulator of progranulin levels and oligomerization

**DOI:** 10.1038/ncomms11992

**Published:** 2016-06-30

**Authors:** Alexandra M. Nicholson, NiCole A. Finch, Marcio Almeida, Ralph B. Perkerson, Marka van Blitterswijk, Aleksandra Wojtas, Basar Cenik, Sergio Rotondo, Venette Inskeep, Laura Almasy, Thomas Dyer, Juan Peralta, Goo Jun, Andrew R. Wood, Timothy M. Frayling, Christian Fuchsberger, Sharon Fowler, Tanya M. Teslovich, Alisa K. Manning, Satish Kumar, Joanne Curran, Donna Lehman, Goncalo Abecasis, Ravindranath Duggirala, Cyril Pottier, Haaris A. Zahir, Julia E. Crook, Anna Karydas, Laura Mitic, Ying Sun, Dennis W. Dickson, Guojun Bu, Joachim Herz, Gang Yu, Bruce L. Miller, Shawn Ferguson, Ronald C. Petersen, Neill Graff-Radford, John Blangero, Rosa Rademakers

**Affiliations:** 1 Department of Neuroscience, Mayo Clinic, Jacksonville, Florida 32224, USA; 2 South Texas Diabetes and Obesity Institute, University of Texas Rio Grande Valley School of Medicine, Brownsville, Texas 78520, USA; 3 Department of Neuroscience, Molecular Genetics, and Psychiatry, University of Texas Southwestern Medical Center, Dallas, Texas 75390, USA; 4 Department of Cell Biology and Program in Cellular Neuroscience, Neurodegeneration and Repair, Yale University School of Medicine, New Haven, Connecticut 06510, USA; 5 Division of Human Genetics, Cincinnati Children's Hospital Research Foundation, Cincinnati, Ohio 45229, USA; 6 Human Genetics Center, School of Public Health, University of Texas Health Science Center at Houston, Houston, Texas 77030, USA; 7 Genetics of Complex Traits, St Luke's Campus, University of Exeter Medical School, University of Exeter, Exeter EX1 2LU, UK; 8 Department of Biostatistics, Center for Statistical Genetics, University of Michigan, Ann Arbor, Michigan 48109, USA; 9 Department of Medicine, University of Texas Health Science Center, San Antonio, Texas 78229, USA; 10 Center for Human Genetics Research, Massachusetts General Hospital, Boston, Massachusetts 02114, USA; 11 Department of Medicine/Cardiology and Cellular & Structural Biology, University of Texas Health Science Center at San Antonio, San Antonio, Texas 78229, USA; 12 Memory and Aging Center, Department of Neurology, University of California, San Francisco, California 94143, USA; 13 Department of Molecular Genetics, Neuroscience, Neurology and Neurotherapeutics, University of Texas Southwestern Medical Center, Dallas, Texas 75390, USA; 14 Department of Neurology, Mayo Clinic, Rochester, Minnesota 55902, USA; 15 Department of Neurology, Mayo Clinic, Jacksonville, Florida 32224, USA

## Abstract

Progranulin (*GRN*) loss-of-function mutations leading to progranulin protein (PGRN) haploinsufficiency are prevalent genetic causes of frontotemporal dementia. Reports also indicated PGRN-mediated neuroprotection in models of Alzheimer's and Parkinson's disease; thus, increasing PGRN levels is a promising therapeutic for multiple disorders. To uncover novel PGRN regulators, we linked whole-genome sequence data from 920 individuals with plasma PGRN levels and identified the prosaposin (*PSAP*) locus as a new locus significantly associated with plasma PGRN levels. Here we show that both PSAP reduction and overexpression lead to significantly elevated extracellular PGRN levels. Intriguingly, PSAP knockdown increases PGRN monomers, whereas PSAP overexpression increases PGRN oligomers, partly through a protein–protein interaction. PSAP-induced changes in PGRN levels and oligomerization replicate in human-derived fibroblasts obtained from a *GRN* mutation carrier, further supporting PSAP as a potential PGRN-related therapeutic target. Future studies should focus on addressing the relevance and cellular mechanism by which PGRN oligomeric species provide neuroprotection.

## Abstract (web summary)

Increasing progranulin (PGRN) levels is a promising approach for treating frontotemporal dementia and other neurodegenerative diseases. Here Nicholson *et al.* show that the prosaposin (PSAP) locus is associated with plasma PGRN levels and demonstrate that PSAP can alter PGRN levels and its oligomerization.

## Graphical abstract

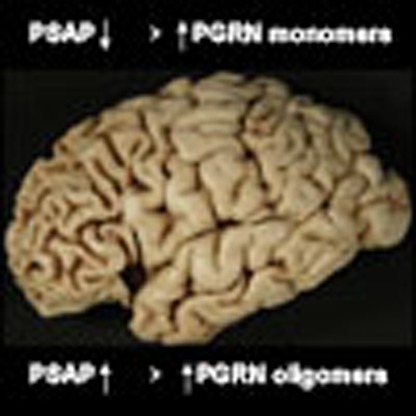

The progranulin protein (PGRN) is a well-studied growth factor widely expressed throughout the human body, with notable expression levels in the bone marrow, immune cells, epithelial cells and the nervous system^1^,^2^,^3^. PGRN is secreted as a full-length glycoprotein comprised of 7.5 tandem repeats of a 10–12 cysteine-containing motif separated by interlinked spacer regions, which can be proteolytically processed into mature 6 kDa granulins^4^,^5^. PGRN's biological function is heterogenous, including cell growth and survival, embryogenesis, inflammatory responses and wound healing^3^,^6^,^7^,^8^. In the brain, PGRN seems to be not only a factor in regulating neuroinflammation, but also a regulator of neurite branching and outgrowth^9^,^10^,^11^,^12^,^13^,^14^.

In 2006, loss-of-function mutations in the PGRN gene (*GRN)* were identified as a common cause of the neurodegenerative disorder frontotemporal dementia (FTD)^15^,^16^. Most FTD-causing *GRN* mutations are autosomal dominant and introduce a premature stop codon through nonsense or frame-shift mutations. Individuals with these mutations are PGRN haploinsufficient due to decay of the mutant messenger RNA (mRNA)^15^,^16^. Importantly, two patients were recently identified with homozygous loss-of-function *GRN* mutations, which caused adult onset neuronal ceroid lipofuscinosis^17^. Neuronal ceroid lipofuscinosis is a lysosomal storage disorder that leads to an accelerated accumulation of lipofuscin^18^, suggesting that adequate levels of PGRN are necessary to maintain proper lysosomal function.

Individuals with *GRN* mutations clinically present with varying symptoms, leading to diagnosis other than FTD in some patients, including Alzheimer's disease, amyotrophic lateral sclerosis and Parkinson's disease^19^,^20^,^21^. While most *GRN* cases show frontotemporal lobar degeneration with TDP-43 pathology at autopsy, Alzheimer's disease pathology has also been reported. A common *GRN* variant, possibly leading to a partial reduction in PGRN levels, has further been suggested as a genetic risk factor for FTD and related neurodegenerative disorders, emphasizing the important role of PGRN in the development of multiple diseases^22^,^23^,^24^,^25^,^26^,^27^. In fact, increasing progranulin levels in both cellular and animal models of FTLD, Parkinson's disease and Alzheimer's disease has been reported as therapeutic^13^,^28^,^29^. PGRN has also been shown to rescue neurite branching and outgrowth in neurons lacking PGRN and protects neurons from TDP-43-related neurotoxicity^13^,^28^. Thus, PGRN upregulation could be a therapeutic approach for multiple neurodegenerative disorders. In recent years, several research groups have conducted studies to identify regulators of PGRN levels. Most notably, the drug suberoylanilide hydroxamic acid was shown to increase *GRN* transcription while alkalizing reagents and vacuolar ATPase inhibitors increased PGRN levels in a post-translational manner^30^,^31^. Nonetheless, no PGRN-modifying therapies are currently available to patients with neurodegenerative disorders.

In this study, we used a genetic approach to identify novel PGRN regulators. Using 920 Mexican-American, non-demented individuals from 20 families, we performed the largest family-based genome-wide association study (GWAS) to date using a combination of whole-genome sequencing (WGS), imputation and PGRN plasma measurements for those individuals. In addition to confirming the chromosome 1p13.3 sortilin (*SORT1*) locus, which was previously implicated in PGRN regulation, this study led to the discovery of a genome-wide significant association between plasma PGRN levels and single nucleotide polymorphisms (SNPs) located on chromosome 10 at the prosaposin (*PSAP*) and cadherin 23 (*CDH23*) locus. Follow-up *in vitro* and *in vivo* studies identified PSAP as a novel regulator of both PGRN levels and PGRN oligomerization. These findings have important implications for the development of PGRN-related therapeutics in FTD and other neurodegenerative disorders.

## Results

### Plasma PGRN level is associated with a chromosome 10q locus

**Figure 1:**
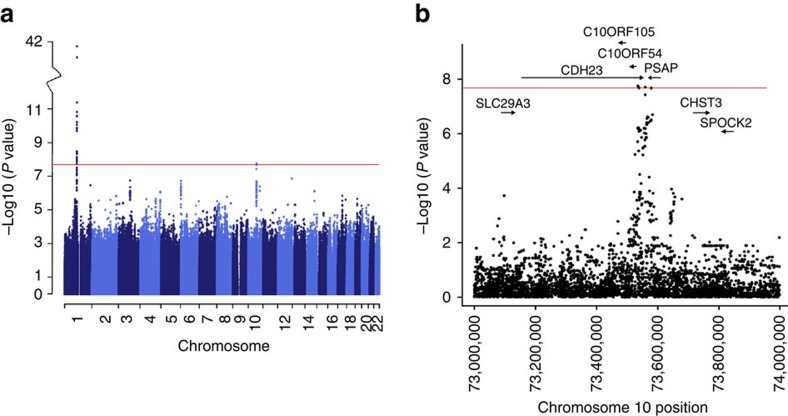
WGS *P* values for the association with PGRN levels. (**a**) Manhattan plot depicting the association results by chromosome in which the −log10 of the *P* values are depicted for each genotyped SNP. (**b**) Regional association data for SNPs located on chromosome 10q21.1–22.2 in which arrows depict the location and directionality of genes within the region. Red horizontal lines indicate the genome-wide significance threshold of *P*=2.7 × 10^−8^.

To identify genetic loci that regulate plasma PGRN levels in non-demented individuals, we conducted a GWAS using WGS data obtained for 920 individuals from 20 Mexican-American families for which plasma PGRN levels were measured by an enzyme-linked immunosorbent assay (ELISA). More than half of the individuals were whole-genome sequenced, while genotypes of the other individuals were generated by imputation (Methods section). The SNP most strongly associated with plasma PGRN levels was rs646776 located near the PGRN receptor *SORT1* on chromosome 1 (linear regression; *P*=8.11 × 10^−42^; Fig. 1a), replicating our earlier findings which were based on a smaller GWAS using roughly 300,000 SNPs (ref. 32). For this SNP, we observed a 0.78 s.d. increment on the average plasma PGRN level for each copy of the minor C-allele. Given the strong *P* values at this locus, quantile-quantile (Q–Q) plots were generated with and without the *SORT1* region (Supplementary Fig. 1). We also identified a second region spanning chromosome 10q21.1–22.2 (linear regression; top SNP rs1867977, *P*=1.39 × 10^−8^) at the prosaposin (*PSAP*) and cadherin 23 (*CDH23*) locus, which reached genome-wide significance (Fig. 1b). SNP rs1867977 showed a 0.28 s.d. decrement on the average plasma PGRN level for each copy of the minor T-allele. To replicate the association with this new locus, we genotyped two additional cohorts of non-demented subjects for which plasma PGRN levels were previously measured^32^,^33^. In replication cohort 1, rs1867977 was significantly associated with plasma PGRN levels (linear regression; *P*=0.007, *n*=269), with each copy of the minor T-allele predicted to decrease the average plasma PGRN level by 0.21 s.d. units (*β*=−0.042). The rs1867977 minor T-allele also correlated with a decrease of the average plasma PGRN level of 0.10 s.d. units per copy in replication cohort 2 (*β*=−0.022); however this change was not statistically significant (linear regression; *P*=0.101, *n*=488).

### rs7869 is a possible variant affecting PSAP levels

**Figure 2:**
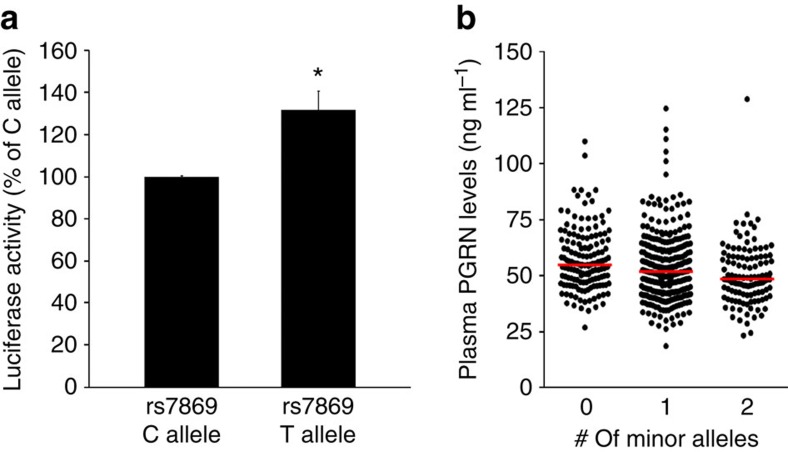
Functional analysis of rs7869-dependent regulation of PSAP and PGRN levels. (**a**) Luciferase activity quantification in HeLa cells transfected with either pMir-REPORT-rs7869C or pMir-REPORT-rs7869T constructs. Graphed values represent the mean±s.e.m. and are expressed as a percent of pMir-REPORT-rs7869C luciferase activity (*n*=13 per group). (**b**) Scatter plot representing plasma PGRN levels in individuals within our initial series expressing the different rs7869 genotypes. Red horizontal lines represent the median plasma PGRN value of each group. *Differs from pMir-REPORT-rs7869C, *P*<0.002 by Student's *t*-test.

Of the genes located within the novel chromosome 10 locus associated with plasma PGRN levels, PSAP was previously implicated as a player in PGRN-related biology due to its similar intra- and extracellular localization, proteolytic processing, receptor binding and its role in lysosomal function^34^. These similarities rendered PSAP the most likely candidate PGRN regulator in this chromosomal region. We therefore queried the WGS data to determine possible *PSAP* functional variants which might affect its levels and/or function. While no *PSAP* coding variants were identified, SNP rs7869 (linear regression; *P*=3.04 × 10^−7^; *D*′=0.854 and *r*^2^=0.384 with rs1867977) resides within the *PSAP* 3′ untranslated region (UTR) (genome.ucsc.edu). SNP rs7869 is a common variant in the San Antonio Family Study (SAFS) cohort (minor allele frequency=48%) and part of a large linkage disequilibrium block. Importantly, when we re-analysed the GWAS conditioned upon the rs7869 genotypes, no residual association was detected (Supplementary Fig. 2), supporting that the observed association in this region is in fact driven by the linkage disequilibrium block represented by rs7869. To establish to what extent the rs7869 minor T-allele might affect *PSAP* expression, we performed mutagenesis on a firefly luciferase construct containing 1,116 bp of the *PSAP* 3′ UTR to create two clones containing either the rs7869 major C- or minor T-allele. HeLa cells showed a significant increase in firefly luciferase activity (mirroring PSAP promoter activity) when transfected with the minor T-allele (Student's *t*-test; *P*<0.002, *n*=13; Fig. 2a). To examine the effect of rs7869 in the context of PSAP directly, we created plasmids containing the human *PSAP* coding sequence along with its endogenous 3′ UTR containing either the rs7869 major C- or minor T-allele (*PSAP*+3′UTR-C and *PSAP*+3′UTR-T, respectively). Both wild-type and mutant rs7869 constructs were transfected into HeLa cells, after which PSAP mRNA and protein levels were quantified. A GFP-expressing vector was co-transfected with these *PSAP*+3′UTR plasmids to serve as a transfection efficiency control. While *PSAP* mRNA levels were not different between cells transfected with either *PSAP*+3′UTR plasmid (Supplementary Fig. 3a), total PSAP protein levels were significantly increased on transfection with *PSAP*+3′UTR-T as compared with *PSAP*+3′UTR-C (Supplementary Fig. 3b). Taken together, these results suggested that rs7869 regulates PSAP protein levels. In our initial GWAS cohort, each copy of the minor T-allele of rs7869 was associated with a decrease in plasma PGRN levels in our GWAS cohort (Fig. 2b). These results reveal a possible reciprocal relationship between PSAP and PGRN levels, in which individuals carrying the rs7869 T-allele have high levels of PSAP and low plasma levels of PGRN.

### Reduction of PSAP increases PGRN levels *in vitro*

**Figure 3:**
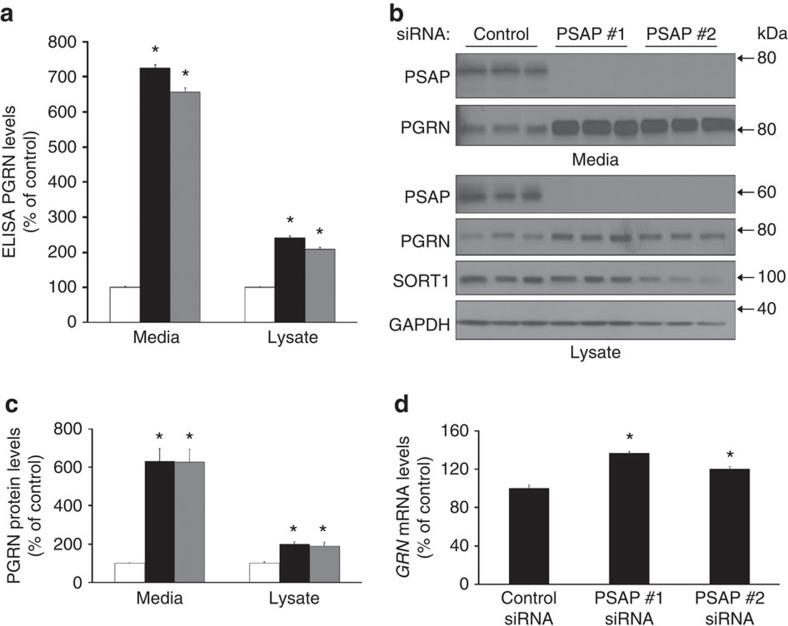
Reducing PSAP levels increases PGRN levels. (**a**) PGRN levels in the media and cell lysates of HeLa cells were measured by the R&D ELISA post siRNA transfection with either a control siRNA (white bars) or PSAP-targeted siRNAs #1 and #2 (black and grey bars, respectively (*n*≥12 per group). (**b**) Immunoblot of media and lysates from HeLa cells transfected with control or PSAP siRNAs. (**c**) Quantification of PGRN immunoreactivity from immunoblots of HeLa cell media and lysates post PSAP knockdown with PSAP siRNA #1 (black bars), #2 (grey bars) or a control siRNA (white bars; *n*=12 per group). (**d**) *GRN* mRNA levels were quantified in HeLa cells post PSAP knockdown (*n*=5 per group). In all graphs, the values represent the mean±s.e.m. *Differs from controls, *P*<0.001 by one-way ANOVA.

Increasing PGRN levels may be a viable therapeutic approach for *GRN* mutation carriers and more generally for FTD and Alzheimer's disease patients. To determine whether reducing *PSAP* levels increases PGRN levels *in vitro*, we transfected HeLa cells with *PSAP*-targeting siRNAs. After 3 days of transfection, *PSAP* knockdown caused a significant increase in PGRN in both cell media and lysates when compared with controls as measured by the R&D Systems PGRN ELISA (one-way analysis of variance (ANOVA); *P*<0.001, *n*≥12; Fig. 3a). More specifically, the PGRN ELISA signal was increased nearly sevenfold in the media, whereas the signal in the lysates was increased over twofold in response to *PSAP* knockdown (Fig. 3a). Similar to the data obtained by ELISA, immunoblotting of the same samples showed that reducing *PSAP* expression increased both intra- and extracellular PGRN levels, with the greatest effects observed in the media of the PSAP siRNA-transfected cells (one-way ANOVA; *P*<0.001, *n*≥12; Fig. 3b,c). These effects on PGRN levels appeared to be independent of the PSAP and PGRN receptor, SORT1 (Fig. 3b). Also, *GRN* mRNA expression was increased in response to *PSAP* knockdown using 20 nM of both PSAP siRNAs and could contribute to the increase in PGRN protein levels (Fig. 3d). However, a dose-response curve using varying PSAP siRNA concentrations still showed a significant increase in media PGRN levels using PSAP siRNA concentrations of 0.1 and 0.05 nM (Student's *t*-tests; *P*<0.001 and *n*=3 for both concentrations) without affecting *GRN* mRNA (Supplementary Fig. 4). These data suggest that reduced levels of PSAP increase PGRN levels in the cell media, at least in part, through a post-translational mechanism.

### PSAP reduction in culture does not alter lysosomal function

**Figure 4:**
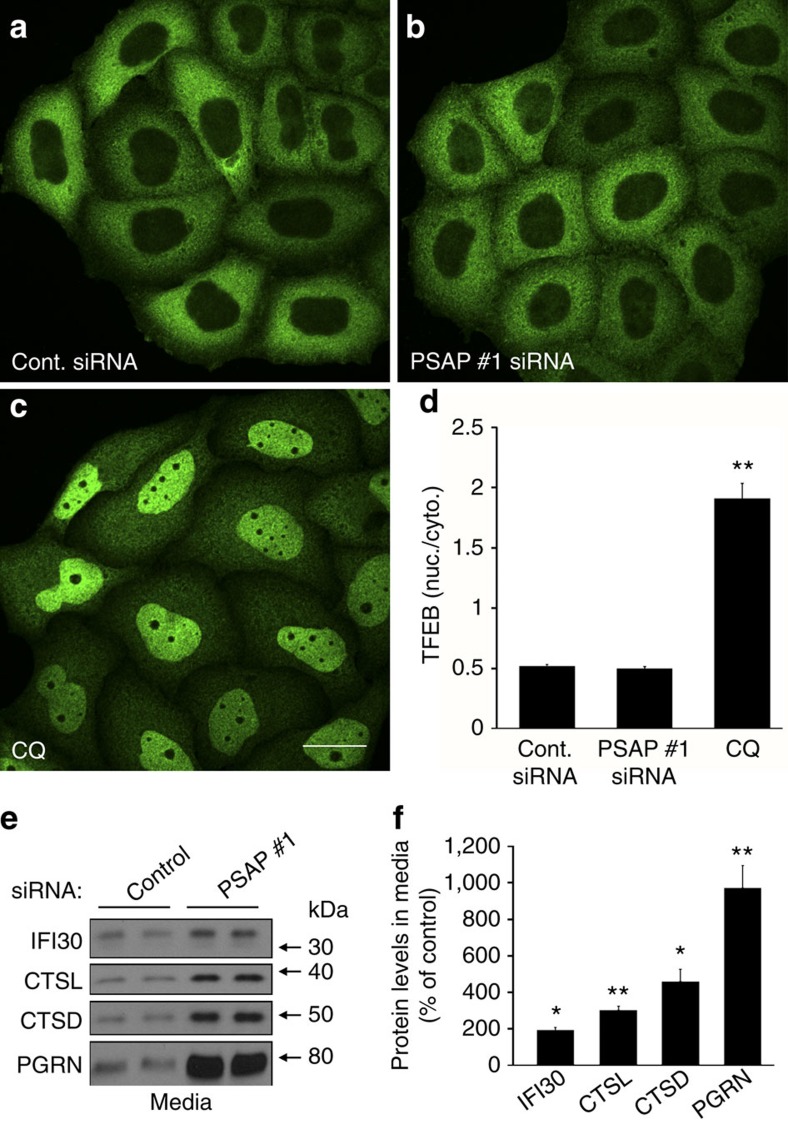
PSAP knockdown does not affect TFEB localization and increases extracellular PGRN more robustly than other lysosomal proteins. (**a**–**c**) GFP immunofluorescence images of HeLa cells that stably express TFEB-GFP. Images were acquired after cells were transfected with 20 nM of either a control (**a**) or PSAP siRNA (**b**), or post treatment with chloroquine (CQ) (**c**). (**d**) Quantification of the effects of siRNA transfection or CQ treatment on the nuclear/cytoplasmic ratio of TFEB-GFP (*n*=21 fields from three independent experiments, totalling ≥70 cells per group). (**e**) Immunoblot of HeLa cell media from 20 nM control or PSAP siRNA transfections. (**f**) Quantification of IFI30, cathepsin L (CTSL), cathepsin D (CTSD) and PGRN protein levels in HeLa cell media post PSAP knockdown (*n*=4 per group). Graphed values represent the mean±s.e.m. *Differs from control-transfected cells, *P*<0.001; **P<0.0005 by one-way ANOVA (**d**) or Student's *t*-tests (**f**). Scale bar, 10 μm.

Complete loss of PSAP or the individual saposins has been shown to cause lysosomal storage disorders. Thus, reducing PSAP levels *in vitro* might alter lysosomal function which, in turn, might influence PGRN levels. Translocation of a transcription factor that specifically recognizes E-box sequences (TFEB) from the cytoplasm to the nucleus has been used to indicate impaired lysosomal function^35^,^36^,^37^. Therefore, we performed PSAP siRNA transfections in HeLa cells that stably express TFEB-GFP. Control and PSAP siRNA transfections showed TFEB-GFP immunofluorescence predominantly in the cytoplasm (Fig. 4a,b, respectively). As a positive control, HeLa cells were treated with chloroquine to disrupt lysosomal function, revealing the majority of TFEB-GFP localization in the nucleus (Fig. 4c). Quantification of TFEB-GFP localization showed a significant increase in the nuclear to cytoplasmic ratio in chloroquine-treated cells, whereas no significant difference in the TFEB-GFP nuclear to cytoplasmic ratio was observed between cells transfected with control or PSAP siRNA (Fig. 4d), indicating that PSAP knockdown in culture does not disrupt lysosomal function.

We also addressed the specificity of PSAP-mediated increases in extracellular PGRN levels. Immunoblots were run of the media samples from control and PSAP siRNA-transfected HeLa cells and were probed with antibodies against other lysosomal proteins known to be secreted, such as cathepsin D, cathepsin L and interferon gamma-inducible protein 30 (IFI30)^38^,^39^,^40^. The immunoreactivity of cathepsin D, cathepsin L and IFI30 was stronger in media from PSAP siRNA-transfected cells as compared to controls (Fig. 4e). However, quantification of the immunoreactive band densities revealed that PSAP reduction had the greatest effect on PGRN as compared to other secreted lysosomal proteins (Fig. 4f).

### Reduction of PSAP increases PGRN levels *in vivo*

**Figure 5:**
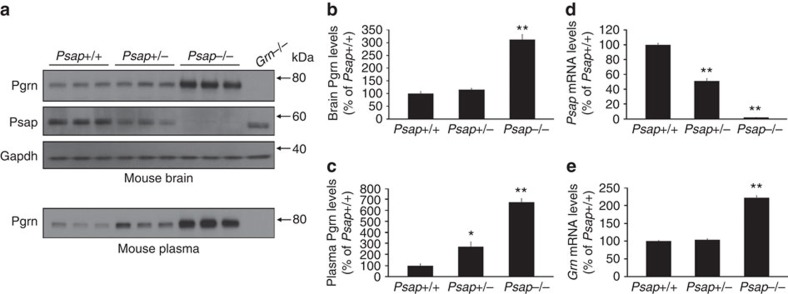
PGRN levels are increased in a mouse model of Psap loss. (**a**) Immunoblot of cortical brain homogenates and plasma obtained from *Psap*+/+, *Psap*+/− and *Psap*−/− mice. *Grn*−/− mouse brain tissue and plasma were used as negative controls. Gapdh immunoreactivity was used to validate equal protein loading. (**b**,**c**) Quantification of Pgrn protein levels in the brains (**b**) and plasma (**c**) obtained from *Psap*+/+, *Psap*+/− and *Psap*−/− mice (*n*=3 per group). (**d**,**e**) Quantification of *Psap* (**d**) and *Grn* (**e**) mRNA levels in *Psap*+/+, *Psap*+/− and *Psap*−/− mouse brains (*n*=3 per group). Values represent the mean±s.e.m. *Differs from *Psap*+/+ mice, *P*<0.05; **P<0.0001 by one-way ANOVA.

To ensure that the increase in PGRN as a result of PSAP reduction could also occur in a brain-relevant cell lines, we repeated the PSAP siRNA transfections in human glioblastoma astrocytoma U251 cells. Similar to what we observed in HeLa cells, decreasing PSAP levels resulted in increased PGRN levels in both the media and lysates of U251 cells (Supplementary Fig. 5a,c). We then assessed whether decreasing PSAP levels upregulates PGRN levels *in vivo* by harvesting cortical mouse brain tissue from wild-type (*Psap*+/+) mice and mice lacking either 1 (*Psap*+/−) or 2 (*Psap*−/−) copies of the *Psap* gene. Immunoblotting of mouse brain tissue and mRNA analyses were used to verify *Psap* loss in *Psap*+/− and *Psap*−/− mice (Fig. 5a,d). Immunoblotting further showed a marked increase in Pgrn levels only in mouse brains completely lacking *Psap* expression as compared with *Psap*+/+ samples (Fig. 5a,b), potentially due to increased *Grn* mRNA levels (Fig. 5e). These data suggest that a 50% loss in *Psap* might not be sufficient to increase Pgrn levels in the brain. However, the protein contained in cortical brain lysates is predominantly intracellular, and since our *in vitro* data suggested that *PSAP* knockdown has the most significant effect on secreted PGRN levels, we also analysed Pgrn levels in mouse plasma. The highest levels of Pgrn were observed in *Psap*−/− mouse plasma (one-way ANOVA; *P*<0.0001 as compared with *Psap*+/+ mice); however, *Psap*+/− mouse plasma Pgrn levels were also significantly increased as compared with *Psap*+/+ (one-way ANOVA; *P*<0.05, *n*=3; Fig. 5a,c), indicating that both partial and complete *Psap* loss are sufficient to increase extracellular Pgrn levels *in vivo*.

### PSAP overexpression changes PGRN levels and oligomerization

**Figure 6:**
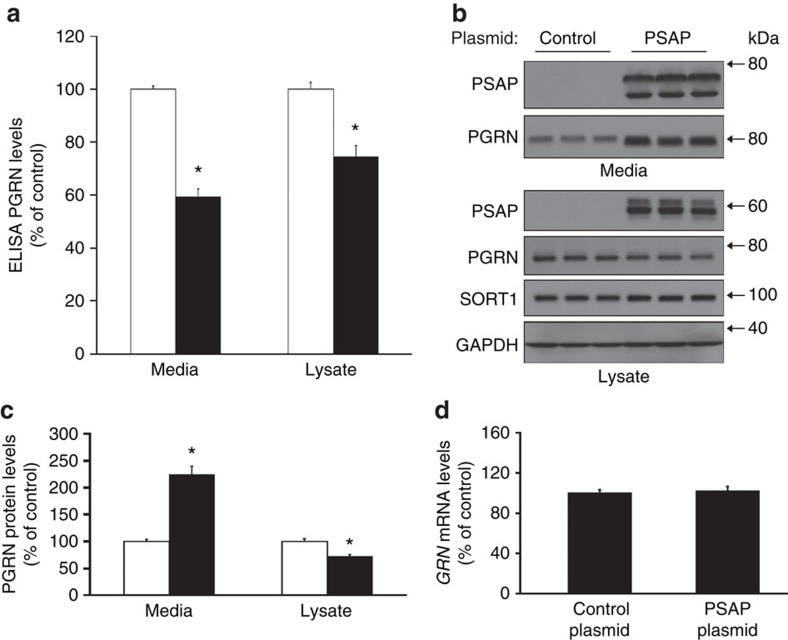
PSAP overexpression yields conflicting PGRN measurements by ELISA and immunoblotting. (**a**) R&D ELISA PGRN measurements in the media and cell lysates of HeLa cells after overexpression of a GFP control (white bars) or PSAP (black bars; *n*≥12 per group). (**b**) Immunoblot of media and lysates from HeLa cells post control plasmid or PSAP overexpression. (**c**) Quantification of PGRN immunoreactivity from immunoblots of HeLa cell media and lysates after transfection with either a control (white bars) or PSAP (black bars) plasmid (*n*≥12 per group). (**d**) Quantification of *GRN* mRNA levels in HeLa cells post PSAP overexpression (*n*≥8 per group). In all graphs, the values represent the mean±s.e.m. *Differs from controls, *P*<0.0001 by Student's *t*-tests.

Based on our GWAS and the fact that PSAP loss results in increased PGRN levels *in vitro* and *in vivo*, we hypothesized that increasing PSAP levels might cause a reduction in PGRN levels. To test this hypothesis, we overexpressed human PSAP in HeLa cells and measured both intra- and extracellular levels of PGRN by ELISA and immunoblotting. PSAP-transfected cells showed a significant decrease of PGRN in both the cell lysate and media compared with controls as measured by ELISA (Student's *t*-tests; *P*<0.0001, *n*≥12; Fig. 6a). These changes were likely post-translational since *GRN* mRNA levels were not affected (Fig. 6d). Immunoblotting was performed on these same samples to validate the changes in PGRN levels post PSAP overexpression. While intracellular PGRN levels were still found to be reduced by PSAP overexpression, immunoblotting unexpectedly revealed a significant increase in extracellular PGRN levels in cells overexpressing PSAP (Student's *t*-test; *P*<0.0001, *n*≥12; Fig. 6b,c). These data conflicted with the ELISA results, which had previously suggested PSAP overexpression to decrease extracellular PGRN levels. Thus, we measured intra- and extracellular PGRN using an alternative PGRN ELISA supplied by Adipogen using HeLa cell conditioned media and lysates with and without PSAP knockdown or overexpression. Consistent with our previous ELISA results, PSAP overexpression resulted in a significant reduction in the PGRN ELISA signal in both the cell media and lysates of PSAP-transfected cells (Supplementary Fig. 6). This indicated that the discrepancy observed between the PGRN ELISA and immunoblotting of PSAP-overexpressing media was not specific to the R&D Systems PGRN ELISA.

**Figure 7:**
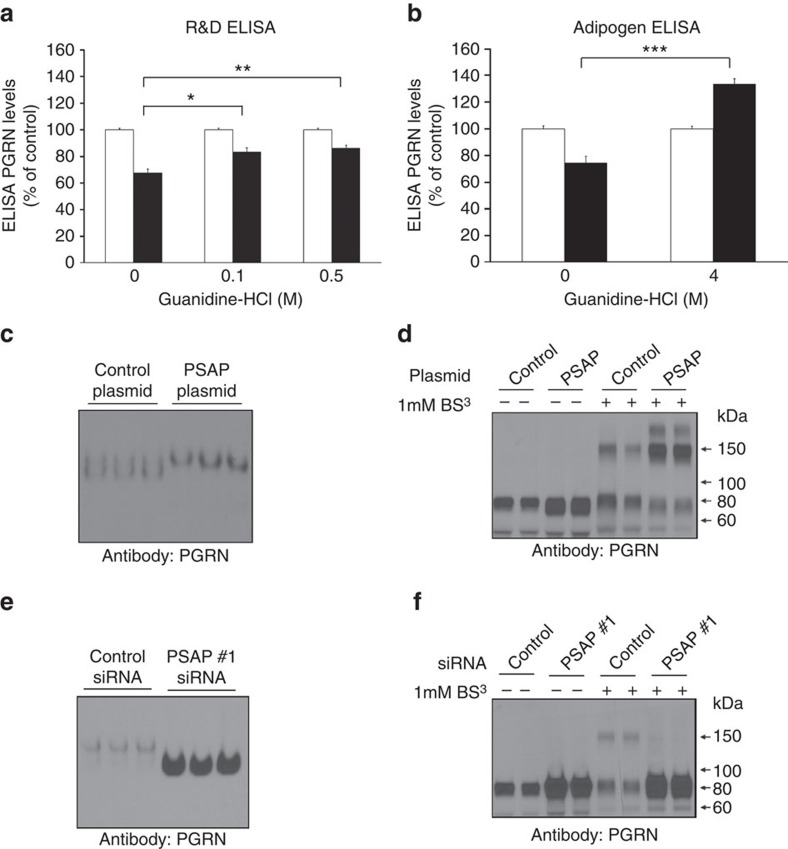
Increasing PSAP levels results in changes in PGRN oligomerization. (**a**,**b**) R&D (**a**) or Adipogen (**b**) PGRN ELISA measurements in HeLa media from control- (white bars) or PSAP-transfected (black bars) cells after a 3-h incubation with or without guanding-HCl (*n*=3 per group). (**c**) Native gel of media from HeLa cells transfected with either a control PSAP overexpression plasmid. (**d**) Immunoblot of media from HeLa cells transfected with either a control or PSAP plasmid that had been treated with (+) or without (−) 1 mM BS^3^. (**e**) Native gel of conditioned media obtained from HeLa cells transfected with a control or PSAP siRNA. (**f**) Immunoblot of control or PSAP siRNA-transfected HeLa cell media after treatment with (+) or without (−) BS^3^ crosslinker. Graphed values represent the mean±s.e.m. *Differs from 0 M guanidine-HCl treatment, *P*<0.05; **P<0.01; ***P<0.0001 by one-way ANOVA (**a**) or Student's *t*-test (**b**).

A significant difference between PGRN measurements by immunoblot versus ELISA is that the proteins are not denatured before ELISA analyses as they are for immunoblotting. A recent study by Nguyen *et al.*^41^ reported that secreted PGRN in its native form exists predominantly as a homodimer^41^. Thus, it is possible that changing PSAP levels alters the ratio of monomeric to dimeric, or possibly higher oligomeric species of PGRN, which might result in decreased epitope recognition by the PGRN ELISAs. To address this possibility, we first denatured proteins in conditioned media from either control- or PSAP-transfected cells using guanidine-hydrochloride (HCl) at varying concentrations before running the PGRN ELISAs. To ensure the guanidine-HCl did not disturb the antibody complexes in the ELISA plate, the final concentration of guanidine-HCl remained 0.5 M or less and all sample results were compared with PGRN standards containing the same final concentration of guanidine-HCl. The R&D Systems ELISA requires the use of undiluted media to detect PGRN, so low guanidine-HCl concentrations (0.1 and 0.5 M) were used for denaturing. In contrast, we used a high guanidine-HCl concentration (4 M) to denature media samples measured on the Adipogen PGRN ELISA plates, which requires the use of diluted samples. Guanidine-HCl treatment significantly increased the PGRN ELISA signal in PSAP-transfected media as compared with PSAP-transfected media without guanidine-HCl treatment (one-way ANOVA; *P*<0.0001; Fig. 7a,b). In fact, incubation of PSAP-transfected media with 4 M guanidine-HCl now showed a significant increase in the PGRN ELISA signal as compared with control-transfected media (Student's *t*-test; *P*<0.001, *n*=3; Fig. 7b), reflective of the immunoblotting data in Fig. 6b. Taken together, these results suggested that PSAP overexpression, in addition to PSAP knockdown, leads to an increase in extracellular PGRN levels in cell culture. Furthermore, PSAP overexpression likely induces the oligomerization of endogenously secreted PGRN.

Further evidence for this hypothesis was obtained by native gel electrophoresis of conditioned media from control- or PSAP-transfected HeLa cells. Compared with media of control-transfected cells, the PGRN detected in media from PSAP-overexpressing cells migrated more slowly (Fig. 7c), suggesting a higher molecular weight. To more accurately determine the molecular size of extracellular PGRN before and after changing PSAP levels, we treated the conditioned media with a chemical crosslinker, bis(silfosuccinimidyl) suberate (BS^3^), that crosslinks primary amines of interacting proteins. Immunoblotting of BS^3^-treated media from cells transfected with a control plasmid revealed PGRN-immunoreactive bands at both ∼80 and ∼160 kDa, suggesting that extracellular PGRN exists in both a monomeric and dimeric form (Fig. 7d)^41^,^42^. Crosslinked media post PSAP overexpression exhibited a subtle decrease in extracellular PGRN monomers while inducing a substantial increase in both PGRN dimers and a higher molecular weight PGRN species not observed in control-transfected media (Fig. 7d). Interestingly, subsequent native gel electrophoresis of conditioned media obtained after PSAP knockdown showed that PGRN migrated more rapidly in these samples compared with control siRNA-transfected cell media (Fig. 7e), suggesting that the predominant PGRN form in this condition was monomeric, a finding further confirmed by BS^3^ crosslinking (Fig. 7f). Similar results were obtained when these experiments were repeated in U251 cells (Supplementary Fig. 5b,d–f).

### PSAP-dependent changes in PGRN occur in *GRN* mutant cells

**Figure 8:**
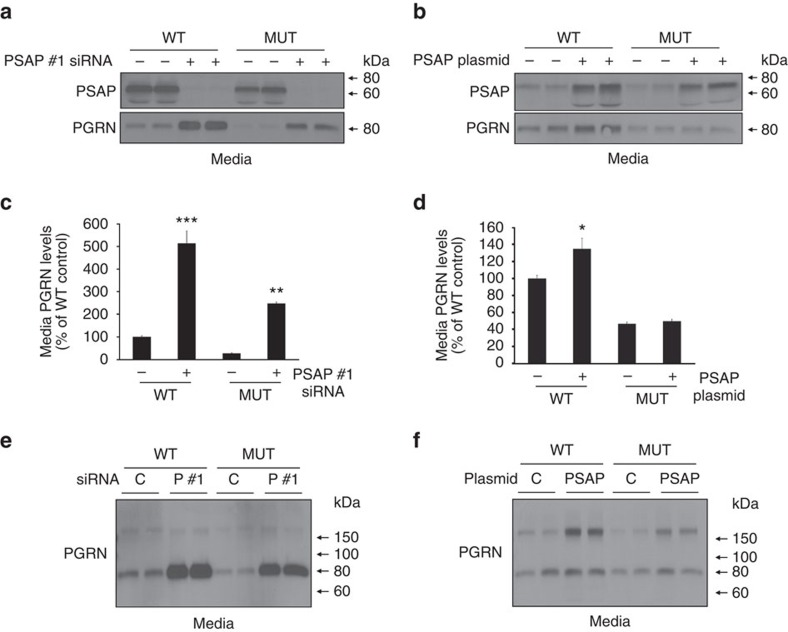
PGRN levels and oligomerization are changed in human fibroblasts derived from individuals with and without *GRN* mutations. (**a**,**b**) Immunoblots of media and lysates obtained from wild type (WT) or *GRN* mutant (MUT) human fibroblasts transfected with either control or PSAP siRNAs (**a**), or with either control or PSAP plasmids (**b**). (**c**,**d**) Quantification of PGRN immunoreactivity in media from WT or *GRN* mutant (MUT) human fibroblasts transfected with either control or PSAP siRNAs (**c**) or with either control or PSAP plasmids (**d**) (*n*=3 per group). (**e**,**f**) Immunoblot of BS^3^-crosslinked media obtained from WT or *GRN* mutant (MUT) human fibroblasts transfected with either control or PSAP siRNAs (**e**) or with either control or PSAP plasmids (**f**). Graphs represent the mean±s.e.m. *Differs from control-transfected cells of the same cell line, *P*<0.05, **P<0.01, ***P<0.001 by one-way ANOVAs.

Our results suggest that both PSAP knockdown and overexpression lead to an increase in total extracellular PGRN levels, albeit different PGRN species, revealing PSAP as a potential novel therapeutic target for individuals carrying *GRN* mutations. To determine to what extent PSAP can rescue extracellular PGRN levels in a cellular model of PGRN loss, we performed PSAP knockdown and overexpression in human-derived fibroblast cells obtained from siblings in which one individual carried a *GRN* mutation. Immunoblotting of control-transfected cells confirmed PGRN loss in the *GRN* mutant cell line as compared with wild-type cells (Fig. 8a,b). Focusing on wild-type cells first, PSAP knockdown or overexpression significantly increased extracellular PGRN levels as compared with control-transfected cells (one-way ANOVA; *P*<0.001, *n*=3; Fig. 8a–d). Moreover, crosslinking experiments confirmed the presence of both monomeric and dimeric PGRN in the media of these fibroblasts and showed that PSAP knockdown led to an increase in monomeric PGRN (Fig. 8e), whereas the level of PGRN dimers was increased upon PSAP overexpression (Fig. 8f). Importantly, in mutant cell media, PSAP knockdown rescued PGRN levels beyond that of wild-type cells (Fig. 8a,c). Crosslinking of this media indicated that the form of PGRN increased by PSAP knockdown was monomeric, similar to what was observed in wild-type cells (Fig. 8e). Although PSAP overexpression was unable to significantly increase the total levels of extracellular PGRN levels in the mutant cell line, crosslinking experiments revealed a marked increase in extracellular PGRN dimers on PSAP overexpression (Fig. 8f).

### PSAP–PGRN interaction

**Figure 9:**
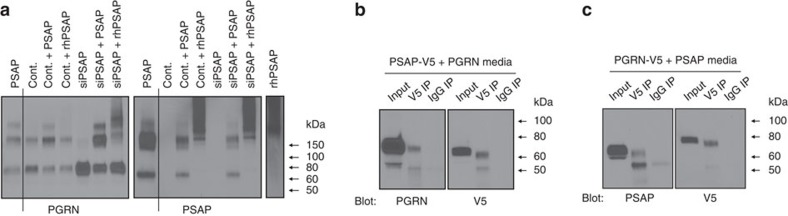
Extracellular PSAP might directly interact with extracellular PGRN. (**a**) Immunoblot of crosslinked conditioned media from HeLa cells transfected with PSAP plasmid (PSAP), control siRNA (Cont.) or PSAP siRNA (siPSAP) mixed together or with rhPSAP. (**b**,**c**) Co-IP assays of conditioned media harvested from HeLa cells co-transfected with PSAP-V5 and untagged PGRN plasmids (**b**) or with PGRN-V5 and untagged PSAP plasmids (**c**).

We next performed media mixture experiments to determine whether exogenous PSAP induces oligomerization of secreted PGRN monomers. To address this question, we harvested conditioned media from control siRNA- or PSAP siRNA-transfected cells. To this media, we added conditioned media from control-transfected cells, conditioned media from PSAP-overexpressing cells, or unconditioned media containing recombinant human PSAP (rhPSAP). These mixtures were incubated for 1 h before conducting BS^3^ crosslinking. When rhPSAP or PSAP-overexpressing media were added to the control media, a slight reduction in PGRN monomers and a slight increase in PGRN dimers and oligomers were observed, suggesting that a small portion of the pre-existing PGRN monomers in the control media formed dimers or oligomers when exogenous PSAP was added (Fig. 9a). More noticeably, when PSAP-overexpressing media or rhPSAP was added to media previously deprived of PSAP, a large fraction of PGRN monomers in this sample were induced to form dimeric and oligomeric species (Fig. 9a). These results indicate that PSAP knockdown-induced PGRN monomer formation is reversible by adding exogenous PSAP, suggesting that PSAP removal does not change the ability of PGRN to form oligomers, but that the presence of PSAP is required for PGRN oligomerization.

Prompted by this finding, we then probed the same immunoblot with a PSAP-specific antibody to determine whether PSAP is also in an oligomeric form in conditioned media. A PSAP-immunoreactive band in crosslinked samples was observed at ∼160 kDa, which is the same molecular weight as one of the PGRN-immunoreactive bands (Fig. 9a). The molecular weight of extracellular PSAP monomers is ∼70 kDa, and extracellular PGRN monomers are ∼80 kDa, so we cannot exclude the possibility that these two proteins are directly interacting to form a heterodimeric species. To determine whether PSAP and PGRN interact extracellularly, immunoprecipitation (IP) assays were performed using conditioned media from HeLa cells that had been co-transfected with PSAP and PGRN in which either the PSAP or PGRN contained a V5 tag at the C-terminus (Fig. 9b,c, respectively). IPs were performed using Protein G magnetic beads that had been bound to either a V5 antibody or an immunoglobulin-G (IgG) control. A portion of the V5-tagged PSAP or PGRN was successfully pulled down with the V5 antibody IP, whereas none was visible in the IgG control (Fig. 9b,c). Furthermore, untagged PGRN protein was pulled down with the PSAP-V5 and untagged PSAP was pulled down with PGRN-V5 (Fig. 9b,c). Taken together, these results identified PSAP as a novel PGRN binding partner and suggested that PSAP induces the oligomerization of PGRN, at least in part, through a direct interaction.

### PSAP does not outcompete PGRN for SORT1 binding

**Figure 10:**
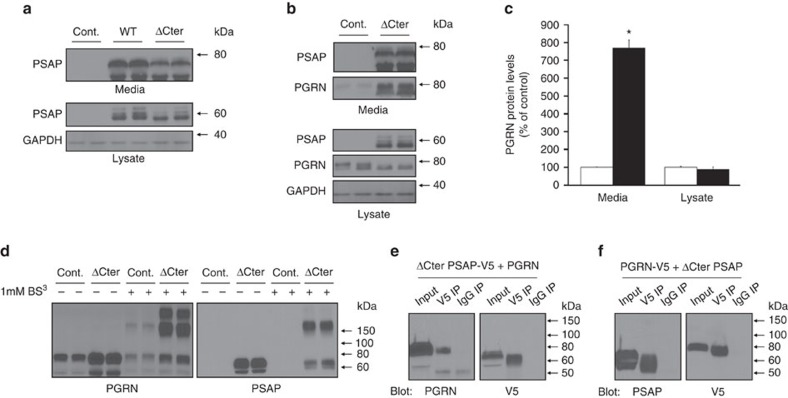
ΔCter PSAP does not affect PSAP-induced changes in PGRN levels or oligomerization. (**a**) Immunoblots of lysates and conditioned media harvested from HeLa cells transfected with either a control (Cont.) plasmid or with wild-type (WT) or ΔCter PSAP plasmids. (**b**) Immunoblots of lysates and conditioned media harvested from HeLa cells transfected with either a control or ΔCter PSAP plasmid. (**c**) Quantification of media and lysate PGRN levels from HeLa cells transfected as described in (**b**) (*n*≥12 per group). (**d**) Immunoblot of media from HeLa cells transfected with either a control or ΔCter PSAP plasmid that had been treated with (+) or without (−) 1 mM BS^3^. (**e**,**f**) Co-IP assays of conditioned media harvested from HeLa cells co-transfected with ΔCter PSAP-V5 and untagged PGRN plasmids (**e**) or with PGRN-V5 and untagged ΔCter PSAP plasmids (**f**). *Differs from control-transfected cells, *P*<0.0001 by Student's *t*-tests.

A feature that is shared between PSAP and PGRN is their ability to bind to the luminal domain of the SORT1 receptor^43^,^44^. In fact, reduction of SORT1 expression has been reported to increase extracellular PGRN levels. Therefore, one potential mechanism by which PSAP overexpression might lead to increased extracellular PGRN is by outcompeting for SORT1 binding. To address this hypothesis, we generated a PSAP expression plasmid lacking the PSAP C-terminus (ΔCter PSAP). PSAP protein lacking this region was previously determined to be inadequate for SORT1 binding^43^. Overexpression experiments in HeLa cells indicated that ΔCter PSAP, like WT PSAP, is overexpressed and localized both intra- and extracellularly (Fig. 10a). Importantly, similar to WT PSAP overexpression (reported in Fig. 6), PGRN levels were significantly increased in the media of HeLa cells transfected with ΔCter PSAP as compared with controls, whereas intracellular levels of PGRN were not changed. (Fig. 10b,c). These findings indicate that the PSAP-induced increase in media PGRN levels on PSAP overexpression are not due to PSAP outcompeting PGRN for SORT1 binding.

In addition to changing PGRN levels, the data provided in Figs 7 and 8 indicated that PSAP overexpression also promotes PGRN homomeric and/or heteromeric oligomerization. To examine whether PSAP's C-terminus is involved in PSAP-mediated PGRN oligomerization, we repeated the BS^3^ crosslinking experiments in HeLa media samples following ΔCter PSAP overexpression. Similar to what we observed with WT PSAP, crosslinked media post ΔCter PSAP overexpression enhanced the formation of higher PGRN oligomeric species with the most prominent immunoreactivity at ∼160 kDa (Fig. 10d). ΔCter PSAP immunoreactivity was also observed in crosslinked media samples at this same molecular weight (Fig. 10d). To determine whether ΔCter PSAP is still able to interact with PGRN, IP assays were performed using conditioned media from HeLa cells that had been co-transfected with ΔCter PSAP and PGRN in which either the ΔCter PSAP or PGRN contained a C-terminal V5 tag (Fig. 10e,f, respectively). Comparable to our observations with WT PSAP, we were able to successfully co-IP PGRN when ΔCter PSAP-V5 was pulled out of the conditioned media (Fig. 10e). In a reciprocal fashion, untagged ΔCter PSAP was observed in the IP sample obtained after IPing for PGRN-V5 (Fig. 10f). Together these results suggest that the C-terminus of PSAP is not required for PSAP-dependent oligomerization of itself or PGRN, nor is it required for the interaction of PSAP and PGRN.

## Discussion

Due to PGRN's neuroprotective properties, identifying proteins or compounds to increase PGRN levels is a key therapeutic avenue, not only for FTD patients with loss-of-function *GRN* mutations, but also for other neurodegenerative diseases. In this study, we identify PSAP as a novel PGRN regulator through an innovative genetics approach linking whole-genome data with plasma PGRN levels in a series of more than 900 non-demented individuals. Using *in vitro* and *in vivo* studies, we show that both PSAP overexpression and knockdown result in increased extracellular PGRN levels; however, the specific PGRN species that were increased is different, with elevated levels of PGRN monomer following PSAP knockdown and increased dimers/oligomers after PSAP overexpression. We further provide evidence that PSAP directly binds to PGRN, establishing PSAP as a novel PGRN binding protein. Our findings highlight a unique layer of complexity when studying proteins or compounds aimed at increasing total levels of PGRN which needs to be considered when identifying new targets for PGRN regulation.

We discovered PSAP as a novel PGRN regulator through association analysis of whole-genome sequence data with PGRN plasma levels, which showed genome-wide significant association with the chromosome 10q21.1–22.2 locus where *PSAP* resides. Genome-wide significant association was also confirmed with the chromosome 1p13.3 region containing *SORT1,* a neuronal receptor for PGRN (and PSAP) which was previously identified as a PGRN regulator in human plasma^32^. Although significant SNPs covered a region containing four genes on chromosome 10q21.1–22.2, *PSAP* was the most obvious candidate to be involved in PGRN regulation because of its similarity to PGRN in structure, function, and neurotrophic properties^34^. In addition, PSAP has a previously established role in both neurodegeneration and brain lysosomal storage disorders^45^.

PSAP is a multifunctional glycoprotein that plays a role in the brain, both intra- and extracellularly. The PSAP precursor protein can be directly secreted into the extracellular matrix where it exhibits neurotrophic properties^46^,^47^. Inside the cell, PSAP is shuttled to the lysosome through an LRP1-dependent secretion-recapture mechanism or by the SORT1 receptor from the trans-Golgi network where it is proteolytically processed into four smaller saposin peptides^48^,^49^,^50^. The saposin peptides (saposins A–D) are critical for lysosomal hydrolase function and subsequent hydrolysis of several glycosphingolipids^48^,^51^. As a result, individuals homozygous for *PSAP* loss-of-function mutations develop one of an assortment of lysosomal storage disorders^52^. In support of a functional role for *PSAP* SNPs in regulating PGRN levels, data mining and *in vitro* functional analyses further identified SNP rs7869 as a potential functional variant regulating PSAP expression. Although it is unknown how rs7869 mediates PSAP protein levels, it is possible that it could alter a microRNA binding site given its location in the 3′ UTR. We recognize that the functional studies performed in cell lines only suggest a role of rs7869 in PSAP and PGRN biology; thus, further examination in primary human cells expressing the different rs7869 variants could further address these questions. In addition, our study does not exclude other *PSAP* variants or other genes within the chromosome 10q region that might contribute to the effects observed in human plasma.

While our initial PGRN ELISA-based measurements and *in vitro* assays suggested an inverse correlation between PSAP and PGRN levels, our follow-up studies unexpectedly indicated that extracellular PGRN levels increased on both PSAP overexpression and knockdown. We observed discrepant findings when comparing PGRN levels as measured by immunoblot and ELISA on PSAP overexpression in cell culture which we were able to attribute, at least in part, to the commercial ELISAs' inability to accurately detect higher molecular weight PGRN species present in these samples. Crosslinking and subsequent immunoblotting indicated that while both PSAP overexpression and knockdown increased total extracellular PGRN levels, PSAP knockdown specifically increased monomeric PGRN levels in cell media, while PSAP overexpression increased PGRN species with dimeric and oligomeric molecular weights. Similar PSAP-mediated effects were observed in U251 glioblastoma astrocytoma cells and in patient-derived fibroblasts from a *GRN* mutation carrier and an unaffected relative, where both PSAP knockdown and PSAP overexpression increased extracellular PGRN levels; yet, in the case of PSAP knockdown, monomeric PGRN species were increased while PSAP overexpression increased dimeric PGRN species. Supporting evidence for the presence of PGRN oligomers was provided by treating media samples with guanidine, by the detection of higher molecular weight PGRN species via native gel electrophoresis, and by media protein crosslinking. The realization that commercially available PGRN ELISA assays may inadequately detect the various PGRN species that exist *in vivo* needs to be considered and might have implications for studies using these ELISAs to identify PGRN regulators or in studies related to the natural history of PGRN levels in human biospecimens and future treatment trials. Equally as important for PGRN-related therapeutics will be to determine the mechanism by which PSAP knockdown and/or overexpression increase extracellular PGRN levels. A likely candidate involved in these processes is the SORT1 receptor utilized by both PGRN and PSAP. The data provided herein exclude the possibility that PSAP overexpression merely out-competes PGRN for SORT1 binding; however, we cannot rule out potential PSAP-related changes in SORT1 localization and/or modification that might have downstream effects on PGRN.

The possible existence of PGRN dimers was first published in 2010 by Okura *et al.*^42^ who detected PGRN-immunoreactive bands at ∼80 and ∼130 kDa from human monocyte-derived macrophage media. A more recent report by Nguyen *et al.*^41^ showed that the majority of secreted and circulating PGRN exists at a dimeric molecular weight. Therefore, it is conceivable that dimeric PGRN is the more biologically relevant and functional PGRN form. Contrastingly, it is also possible that monomeric PGRN turnover is more rapid, and thus exists at levels below the detection limits in these fluids. Additional research is required to further elucidate the PGRN species that exist in various fluids and tissues, and to address to what extent the different PGRN forms, either monomeric or oligomeric, might be responsible for PGRN-dependent neuroprotection. This is crucial since both PSAP knockdown and overexpression are able to increase total extracellular PGRN levels in human-derived cell lines, but only one of these strategies may be a beneficial therapeutic avenue. Second, it will be critical to further characterize the molecular mechanisms behind PSAP-induced PGRN oligomerization. In this respect, it is important to note that PGRN might form a complex with other proteins, producing an extracellular PGRN species identified at a molecular weight similar to a PGRN homodimer. Since the molecular weight of fully glycosylated PSAP and PGRN is similar (∼70 and ∼80 kDa, respectively), PSAP–PGRN heterodimers might be easily interpreted as PGRN homodimers. Our current data *in vitro* already indicates that at least a subset of PGRN is bound to PSAP and that the C-terminus of PSAP is not required for this interaction. If in fact a subset of dimeric PGRN is composed of PSAP–PGRN heterodimers, this may explain why loss of PSAP in cell culture caused an increase in the monomeric form of extracellular PGRN. On the other hand, we cannot exclude that extracellular PSAP regulates the formation of PGRN homodimers or oligomerization with other proteins. Furthermore, it remains unknown whether the PSAP/PGRN interaction is required for the effect of PSAP on PGRN levels. If future studies are able to determine the specific region of PSAP required for PSAP–PGRN binding, it may be possible to elucidate the relationship between these two phenomena.

Finally, we assessed the *in vivo* consequence of *Psap* deficiency using a knockout mouse model previously generated to study lysosomal storage disorders. In support of our human plasma and cell culture data, we observed an increase in Pgrn protein levels in brain tissue from *Psap* knockout mice, but only in the full knockout and not in heterozygous *Psap*+/− mice. Importantly, this effect on intracellular Pgrn levels is not unique to *Psap* knockout mice since a previous report showed that ablation of another critical lysosomal protein, cathepsin D, also resulted in upregulation of Pgrn and other lysosomal proteins, likely due to lysosomal enlargement or lack of lysosomal-dependent degradation^53^. The intracellular changes in Pgrn and other lysosomal proteins may, therefore, reflect a dysfunction of the lysosomes in this model system, and are independent from PSAP's extracellular effects on PGRN levels revealed in our studies. This would be supported by the fact that we did not observe an increase in brain intracellular Pgrn levels in mice heterozygous for Psap loss. In contrast, mice with 50% loss of Psap did show a marked increase in plasma Pgrn levels, indicating that partial Psap reduction is sufficient to increase extracellular Pgrn *in vivo*. This is in line with the TFEB translocation assay that we performed in HeLa cells after PSAP knockdown, which excluded the possibility that PGRN is increased in response to PSAP reduction due to PSAP-mediated lysosomal dysfunction. These findings are exciting and suggest that while complete loss of PSAP has overall detrimental outcomes and results in lysosomal storage disease, it is possible that partial loss of PSAP might have beneficial effects. In fact, no reports have been published to describe health deficits in individuals heterozygous for *PSAP* mutations. Thus, mildly reducing PSAP levels might be a promising therapeutic for increasing extracellular PGRN.

Taken together, this study is the first to identify PSAP as a PGRN regulator. Our functional assessment of PSAP-mediated changes in PGRN also furthered our understanding of PGRN-related methodologies and biology for consideration in future PGRN assessments. First, our data revealed that the current commercially available ELISAs commonly used for PGRN measurements in human fluids do not detect all PGRN forms. This has potential consequences for PGRN-related therapeutics in which PGRN ELISAs are being proposed as a tool to monitor the efficacy of PGRN-targeting pharmacological agents. Second, our findings are the first to exemplify a PGRN regulator that mediates not only total PGRN levels, but also PGRN oligomeric composition. A better understanding of the involvement and functional mechanisms of different PGRN oligomeric species will be greatly important in the PGRN field.

## Methods

### Initial subjects cohort and whole-genome sequencing

The WGS data was kindly shared by the T2D-GENES project (unpublished data). T2D-GENES is a large collaborative study composed of 1,039 individuals that where whole-genome sequenced or directly imputed as described below. Those individuals were drawn from 20 large Mexican-American pedigrees with 22–86 individuals per family over 3–5 generations (48.9% males). All participants live in the great San Antonio area, Texas, USA and are all participants of the SAFS project in which all patients provided informed consent^54^. The use of human samples by T2D-GENES and the SAFS project were approved and follow all guidelines enforced by the University of Texas Rio Grande Valley Institutional Review Board.

Initially, a set of 586 individuals was sequenced using a sequence-by-ligation method by Complete Genomics Inc. (Mountain View, CA). The sequencing paired-end reads of 70 bp were mapped in the human genome reference (V 37.2) with a mean coverage of 60x. The genetic variants were called by Complete Genomics Inc. using their proprietary dedicated software (version 2.0.3.1). The rate of genotype discordance between called alleles in the WGS data and a previously generated GWAS was between 0.2 and 0.6% for each sequenced sample. The WGS data generated on the 586 individuals was then used in conjunction with the known pedigree relationship and the previously generated genome-wide DNA chip data for an effective offspring imputation using MaCH software^55^,^56^. A set of 453 individuals were imputed with high confidence giving a final total sample size of 1,039 densely genotyped individuals. In total 21.5 million single nucleotide variants were identified of which 67% were rare with a minor allele frequency below 1% (Supplementary Tables 1–3). This proportion is expected and highlights the power of large pedigrees for the assessment of rare variants in phenotypes of interest. Please note that only a subset of these individuals (*n*=920) were included in the GWAS presented in this study because only these individuals also had plasma samples available for PGRN measurement.

### Replication subjects cohorts and sequencing

Chromosome 10 SNPs rs7869 and rs1867977 genotyping was performed in replication cohorts 1 and 2 by either Sanger sequencing or the Sequenom MassArray iPLEX platform (San Diego, CA) and Typer 4.0 software. Replication cohort 1 includes 269 non-demented individuals recruited into the Mayo Clinic Study of Aging for which details are described elsewhere^33^,^57^,^58^. Replication cohort 2 was also previously published and consists of 488 non-demented individuals recruited at the Mayo Clinic Jacksonville^32^. Individuals included in the replication cohorts gave written consent with approval from the Mayo Clinic and Olmsted Medical Center Institutional Review Boards.

### PGRN ELISAs

Plasma PGRN levels of the replication subjects cohorts were available from previous studies^32^,^33^. To determine the PGRN levels in plasma samples from the subjects in the initial cohort or in cell culture samples, we used the Quantikine Human Progranulin Immunoassay (R&D Systems, Minneapolis, MN) per the manufacturer's instructions using undiluted samples analysed in duplicate. ELISA plates were analysed with samples randomized based on family number, age at draw, gender and years of plasma storage. Supplementary Fig. 7 shows the PGRN plasma distribution in our population. Cell culture conditioned media were also analysed with the Human Progranulin ELISA Kit from Adipogen Inc. (Seoul, Korea) in which the sample was diluted 1:50 in the provided dilution buffer and analysed in duplicate. Each kit's provided recombinant human PGRN was used as a standard. A subset of PGRN ELISAs was carried out using media samples denatured with guanidine-HCl. For the R&D ELISA, 10 μl of 1 M guanidine-HCl was added to 90 μl of either control- or PSAP-transfected media and this was left to incubate at room temperature for 3 h before loading into the ELISA plate. For the Adipogen PGRN ELISA, 50 μl of 8 M guanidine-HCl was added to 50 μl of media from either control- or PSAP-transfected cells and incubated for 3 h at room temperature. Post incubation, the samples were further diluted 1:25 in the provided dilution buffer before loading into the ELISA plate. All guanidine-treated samples were compared with the provided PGRN standards that also contained the same final concentration of guanidine-HCl.

### Cell culture and transfections

HeLa cells (purchased from American Type Culture Collection, Manassas, VA) were cultured in Eagle's minimum essential medium supplemented with 10% foetal bovine serum (FBS) and 1% penicillin/streptomycin (pen/strep). U251 cells (a generous gift from Dr Jenkins of Mayo Clinic Rochester) were maintained in Dulbecco's modified eagle medium supplemented with 10% FBS, 1% pen/strep and 1% L-glutamine. Human fibroblast cell lines were obtained from the University of California San Francisco and were also cultured in Dulbecco's modified eagle medium supplemented with 10% FBS, 1% pen/strep and 1% non-essential amino acids. All lines were maintained at 37 °C, 5% CO_2_.

*PSAP* gene knockdown was achieved by reverse transfection of 150,000–250,000 cells per well in a 6-well culture dish using siRNA1 (target sequence 5′-AAGAAAUACUCGACGCUUU-3′, J-003694-17, GE Dharmacon, Lafayette, CO) or siRNA2 (target sequence 5′-CGACAUAUGCAAAGACGUU-3′, J-003694-18, GE Dharmacon) designed against the coding region. A non-targeting siRNA (target sequence 5′-UGGUUUACAUGUCGACUAA-3′, D-001210-05, GE Dharmacon) was used as a control. Briefly, RNA oligonucleotides were incubated in 500 μl media with Lipofectamine RNAiMax (Life Technologies) transfection reagent per the manufacturer's protocol and added to each well of 6-well culture dishes, after which the cells were plated in growth medium free of antibiotics. siRNAs were used at a final concentration in each well of 0.01–20 nM. For complementary DNA (cDNA) transfections, the cells were plated one day prior in 6-well culture dishes at 200,000–300,000 cells per well. Cells were transfected the following day with plasmid DNA (2 μg plasmid for single transfections and 1 μg of each plasmid for co-transfections) using the Lipofectamine 2000 transfection reagent (Life Technologies, Grand Island, NY) or X-tremeGENE HP DNA Transfection Reagent (Roche Life Science, Indianapolis, IN) per the manufacturer's instructions. Of note, all cell transfections were completed in growth medium supplemented with 5% FBS for all experiments in which media was collected for immunoblotting to reduce interference of the bovine serum albumin contained in the serum supplement. For overexpression experiments, the pAAV empty vector, eGFP-pAAV or pEGFP-N1 was used as a control. Untagged and V5-tagged PSAP-pAAV constructs were generated by PCR-amplifying the coding sequence of PSAP from PSAP-pCMV-XL5 (Origene) using primers to maintain the stop codon or to add a C-terminal V5 tag. Untagged and V5-tagged C-terminal deletion (ΔCter) PSAP constructs were generated using primers to remove amino acids 491–526 from the human PSAP protein sequence. Primer sequences are included in Supplementary Table 4.

### Immunoblotting

Media samples for analysis were prepared by harvesting the media 3 days post transfection and removing debris by centrifugation for 5 min at 3,824*g* at 4 °C. Lysates were prepared by harvesting 3 days post transfection in radioactive IP assay buffer (Boston BioProducts) supplemented with protease/phosphatase inhibitors (Thermo Scientific, Waltham, MA). Undiluted media and lysate samples were mixed with an equivalent volume of Novex sample buffer (Life Technologies) supplemented to 5% β-mercaptoethanol. Proteins were denatured by heating at 95 °C for 5 min before loading into SDS-polyacrylamide gels (Life Technologies), transferred to Immobilon membranes (Millipore), and immunoblotted with the primary antibody. The next day, blots were incubated with an HRP-conjugated secondary antibody (1:5,000; Promega) and bands were detected by enhanced chemiluminescence using Western Lightning *Plus*-ECL reagents (Perkin Elmer, Waltham, MA). Full blot images of all immunoblots included in Figs 3, 4, 5, 6, 7, 8, 9, 10 can be visualized in Supplementary Figs 8–15.

The following primary antibodies were used: rabbit anti-PGRN (1:1,000; Life Technologies), rabbit anti-PSAP (1:1,000; ProteinTech, Chicago, IL), rabbit anti-Psap^59^ (1:20,000; a generous gift from Dr Sun, Cincinnati Children's Hospital Medical Center), mouse anti-SORT1 (1:1,000; R&D Systems), goat anti-V5 (1:10,000; Novus Biologicals, Littleton, CO), mouse anti-GFP (1:20,000; Millipore, Billerica, MA), sheep anti-IFI30 (1:200; R&D Systems), goat anti-cathepsin D (1:200; R&D Systems), goat anti-cathepsin L(1:200; R&D Systems) and mouse anti-GAPDH (1:500,000; Meridian Life Science, Cincinnati, OH). The polyclonal anti-progranulin antibody (used at 1:10,000) was generated by injecting into rabbits a carboxyl-terminally amidated peptide (linker 1, (C)TLLKKFPAQKTNRAVSL, amino acid residues 185–202) through an N-terminally introduced cysteine residue to maieimide-activated keyhole limpet haemocyanin. All procedures were done according to the institutionally standardized rabbit immunization protocol that was reviewed and approved by the Institutional Animal Care and Use Committee (IACUC) at UT Southwestern following AAALAC guidelines.

### TFEB-GFP localization

PSAP knockdown was performed as described above using a final concentration of 20 nM siRNA. Transfection complexes were gently added to each well of a 24-well tissue culture plate containing HeLa cells stably expressing TFEB-GFP (ref. 36). Cells were incubated at 37 °C for 72 h. Where indicated, the incubation of cells with 50 μM chloroquine for 12 h at 37 °C was performed as a positive control that perturbs lysosome function and causes robust TFEB-GFP translocation to the nucleus^36^. Cells were fixed in 4% paraformaldehyde and immunocytochemical analysis was performed using an anti-GFP antibody (Roche) for TFEB-GFP detection and 4′,6-diamidino-2-phenylindole (1 mg ml^−1^; Life Technologies) to stain nuclei.

Spinning disc confocal microscopy was performed with a Nikon Ti-E Eclipse inverted microscope (equipped with a × 40 Plan Apochromat (NA 1.0) oil immersion objective) and a spinning disk confocal scan head (CSU-X1; Yokogawa, Tokyo, Japan) driven by Volocity (Improvision; Perkin Elmer, Waltham, MA) software. Seventy to eighty TFEB-GFP-expressing cells were analysed per experiment and the ratio of nuclear/cytoplasmic TFEB in three separate experiments was quantified using CellProfiler software^60^. To this end, nuclei were identified by 4′,6-diamidino-2-phenylindole staining and nuclear edges were uniformly expanded (10 pixels) to form a halo that defined the surrounding cytoplasmic compartment in each cell. This enabled us to measure the mean intensities of the nuclear and cytoplasmic intensities and subsequently calculate the TFEB nuclear/cytoplasmic ratios on a cell-by-cell basis.

### Mouse tissue harvest and sample preparation

Male *Psap*+/+, *Psap*+/− and *Psap*−/− mice^61^,^62^ were subjected to CO_2_ narcosis at 3 weeks of age and perfused with 10–15 mls saline solution. Before perfusion, the blood was collected from the hepatic portal vein and transferred into a tube with 20 μl of 0.5 M EDTA. Plasma was separated by centrifugation at 2,300*g* at 4 °C for 10 min, and stored at −80 °C. On use for immunoblotting, the plasma was thawed and diluted 1:50 in PBS, after which the sample was denatured in an equivalent volume of Novex sample buffer and loaded into SDS-polyacrylamide gels (Life Technologies). Brain tissue was extracted and stored at −80 °C before use. For immunoblotting experiments, ∼50 mg cortical brain tissue was homogenized in 200 μl radioactive IP assay buffer (Boston BioProducts) supplemented with protease/phosphatase inhibitors (Thermo Scientific, Waltham, MA). Samples were subjected to centrifugation at 4 °C for 5 min at 20,817*g* and the supernatant was transferred to a fresh tube. Protein concentrations were determined by the Pierce Bicinchoninic Acid Protein Assay (Thermo Scientific) per the manufacturer's instructions using known concentrations of bovine serum albumin as standards. Lysates were prepared at a concentration of 2 mg ml^−1^ protein in Novex sample buffer before denaturing by heating 5 min at 95 °C. All animal protocols and procedures were in compliance and approved by the IACUC at Cincinnati Children's Hospital Medical Center.

### Crosslinking assays

HeLa cells were transfected as described above and media samples were prepared for crosslinking as follows. Two days after transfection, the media were changed to serum-free overnight. The following day, the media were harvested and centrifuged for 5 min at 3,824*g* at 4 °C to remove cell debris. For media mixture experiments, or for media mixed with 4 μg rhPSAP prepared in serum-free unconditioned media, an equivalent volume of each was added to a 15-ml conical tube and incubated for 1 h at 37 °C before clearing. Two 500 μl aliquots of each sample were concentrated to ∼25 μl using Amicon Ultra 0.5 ml concentrators (50 kDa cutoff; Millipore) for 10 min at 14,000*g* at room temperature. Crosslinking was performed using BS^3^ (Thermo Scientific) prepared in water to 25 mM. BS^3^ was added to concentrated conditioned media at a final concentration of 1 mM (or an equivalent volume of water for an uncrosslinked control) and incubated for 1 h at room temperature. The reaction was quenched for 15 min at room temperature by adding 500 mM Tris-HCl, pH 7.4 to a final concentration of 50 mM before denaturing in an equivalent total volume of sample buffer as described above.

### Mutagenesis and luciferase activity assays

The pMirTarget firefly luciferase expression vector containing the *PSAP* 3′ UTR was purchased from Origene (Rockville, MD). Introduction of the rs7869 rare variant SNP was achieved by cloning the *PSAP* 3′ UTR into pcDNA3.1 before utilizing the QuickChange site-directed mutagenesis protocol (Agilent Technologies, Santa Clara, CA). Primer information is available in Supplementary Table 4. The presence of the mutant was verified by direct sequencing and the *PSAP* 3′ UTR was cloned back into the pMirTarget firefly luciferase expression vector. Wild-type or mutant firefly luciferase constructs (1 μg) were co-transfected with pRL-CMV-renilla luciferase (100 ng) into HeLa cells that were plated 1 day prior. The next day, firefly and renilla luciferase activities (LA_F_ and LA_R_, respectively) were measured in the same samples in triplicate using the Dual-Luciferase Reporter Assay System (Promega, Madison, WI) and a Veritas microplate luminometer. Relative luciferase activity was quantified as LA_F_/LA_R_.

Constructs containing the *PSAP* coding sequence and 3′ UTR were generated by first cloning the *PSAP* coding sequence from PSAP-pAAV into the pcDNA3.1(+) vector (PSAP-pcDNA3.1). We then PCR-amplified the PSAP 3′UTR from human cDNA and inserted it into the PSAP-pcDNA3.1 plasmid using an internal BamHI restriction site. Introduction of the rs7869 rare variant SNP was achieved by the QuickChange site-directed mutagenesis protocol and primers (Supplementary Table 4) as described above.

### RNA isolation and quantitative PCR

Total RNA was extracted from transfected HeLa cells or from 40 mg of mouse brain cortical tissue using the RNeasy Plus Mini Kit (Qiagen) or PureLink RNA mini kit (Life Technologies), respectively. Using 300 ng RNA per sample as a template, a reverse transcription reaction was performed using the Superscript III system (Life Technologies). Real-time quantitative PCR using an ABI7900 was performed in triplicate for each sample using Life Technologies human gene expression probes for *GRN* (Hs00963703_g1), *PSAP* (Hs01551096_m1), *GAPDH* (Hs00266705_g1) and *RPLP0* (Hs00420895_gh), as well as mouse gene expression probes for *Grn* (Mm01245914_gl), *Psap* (Mm00478338_ml), *β-Actin* (Mm01205647_gl) and *Gapdh* (Mm99999915_gl). A *GFP* probe (Mr04097229_mr, Life Technologies) was used to ensure equal transfection for the indicated co-transfection experiments. Results were analysed using SDS software version 2.2 and relative quantities of *GRN* or *PSAP* mRNA were determined.

### Immunoprecipitation

HeLa cells were co-transfected with V5-tagged wild-type or ΔCter PSAP-pAAV and untagged PGRN-pCMV-SPORT6, or with V5-tagged PGRN-pcDNA6 and untagged wild-type or ΔCter PSAP-pAAV. Two days post transfection, cell media was changed to serum-free and harvested for IP 24 h later. Briefly, 10 ml media was concentrated in a Amicon Ultra 15 ml concentrator (50 kDa cutoff) by centrifuging for 8 min at 3,220*g*. Samples were brought up to 1 ml with un-supplemented EMEM and 500 μl was used for IPs using Protein G-coated Dynabeads (Life Technologies). The Dynabeads were pre-incubated with 2 μg anti-V5 antibody (ref. 46–0705, Life Technologies) or 2 μg of a mouse whole-molecule IgG control (Jackson Laboratories) before IP. IP complexes were removed from the beads by incubation in the provided elution buffer for 10 min, after which an equivalent amount of Novex sample buffer (5% β-mercaptoethanol) was added and samples were heated 5 min at 95 °C before removing the beads.

### Statistics

For the genetic association analyses in the initial and replication subjects cohorts, plasma PGRN concentration was normalized using an inverse Gaussian normalization and the ‘genotype x trait' association had been determined using the additive variance component test as implemented in SOLAR (ref. 63). To correct for possible confounding factors we used sex and age as covariates in addition to the first three Principal Components. The components were pre-calculated using the genotypic dosage of the founders and unrelated individuals in our pedigree and were used to correct for unknown latent population stratification.

For all other experiments in which only two groups were compared, significance was measured using a two-tailed Student's *t*-test. For analyses involving more than two groups, GraphPad Prism 5.04 (GraphPad Software) was utilized to perform a one-way ANOVA followed by the Tukey's multiple comparison *post-hoc* test.

### Data availability

The T2D-GENES whole-genome sequence data set used in this genetic association study has been deposited in dbGAP with the primary accession code: phs000462.v1.p1. Detailed demographic information is only available on request to the corresponding author since this information could compromise research participant privacy or consent. Plasma PGRN data are also available from the corresponding author on request. The authors declare that all other supporting data supporting the findings of this study are available within the article and its Supplementary Information Files or are available from the corresponding author on request.

## Additional information

**How to cite this article:** Nicholson, A. M. *et al.* Prosaposin is a regulator of progranulin levels and oligomerization. *Nat. Commun.* 7:11992 doi: 10.1038/ncomms11992 (2016).

## Supplementary Material

Supplementary Information Supplementary Figures 1-15 and Supplementary Tables 1-4
