# Supplementary material for: Prosaposin is a regulator of progranulin levels and oligomerization
Source: Nat Commun. 2016 Jun 30;7:11992. doi: 10.1038/ncomms11992 (PMC4931318; doi:10.1038/ncomms11992)
Supplement: Supplementary Information — Supplementary Figures 1-15 and Supplementary Tables 1-4 [file ncomms11992-s1.pdf]

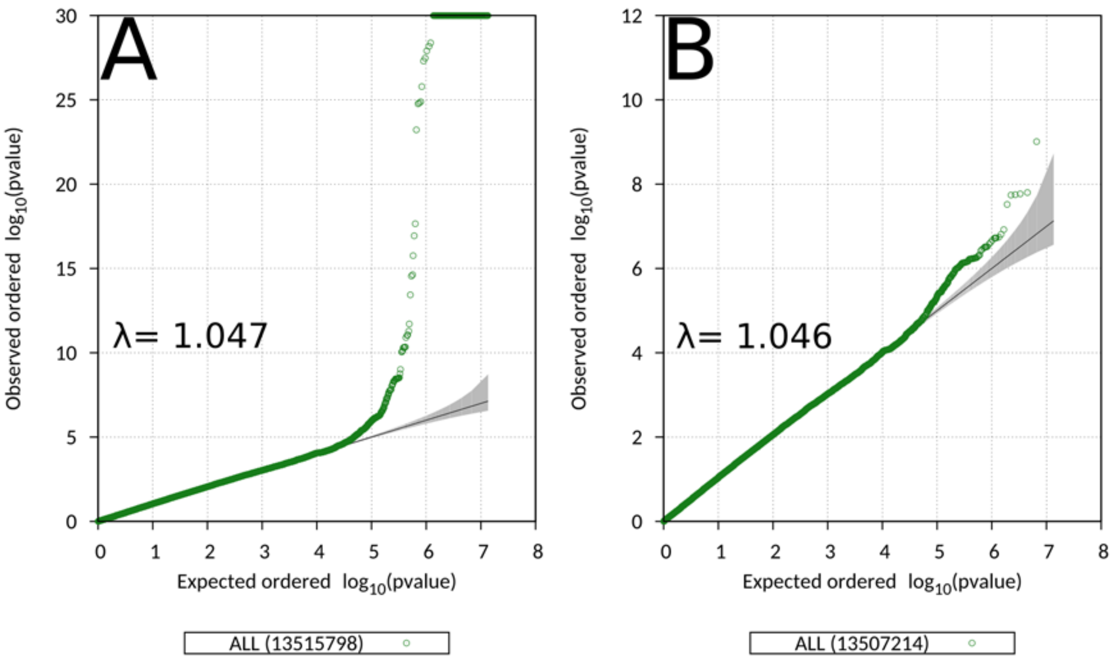

Supplementary Figure 1. Quantile-quantile (Q-Q) plots. (Panel A) Q-Q plot graphical representation using all SNPs (n= 13,515,798) including the region on chromosome 1 including *SORT1* which was previously shown to be associated with plasma PGRN levels. **(Panel B)** Q-Q plot graphical representation excluding the region on chromosome 1 with the strongest association (number of SNPs = 13,507,214). Associated inflation factors (λ) were calculated and shown in each Q-Q plot.

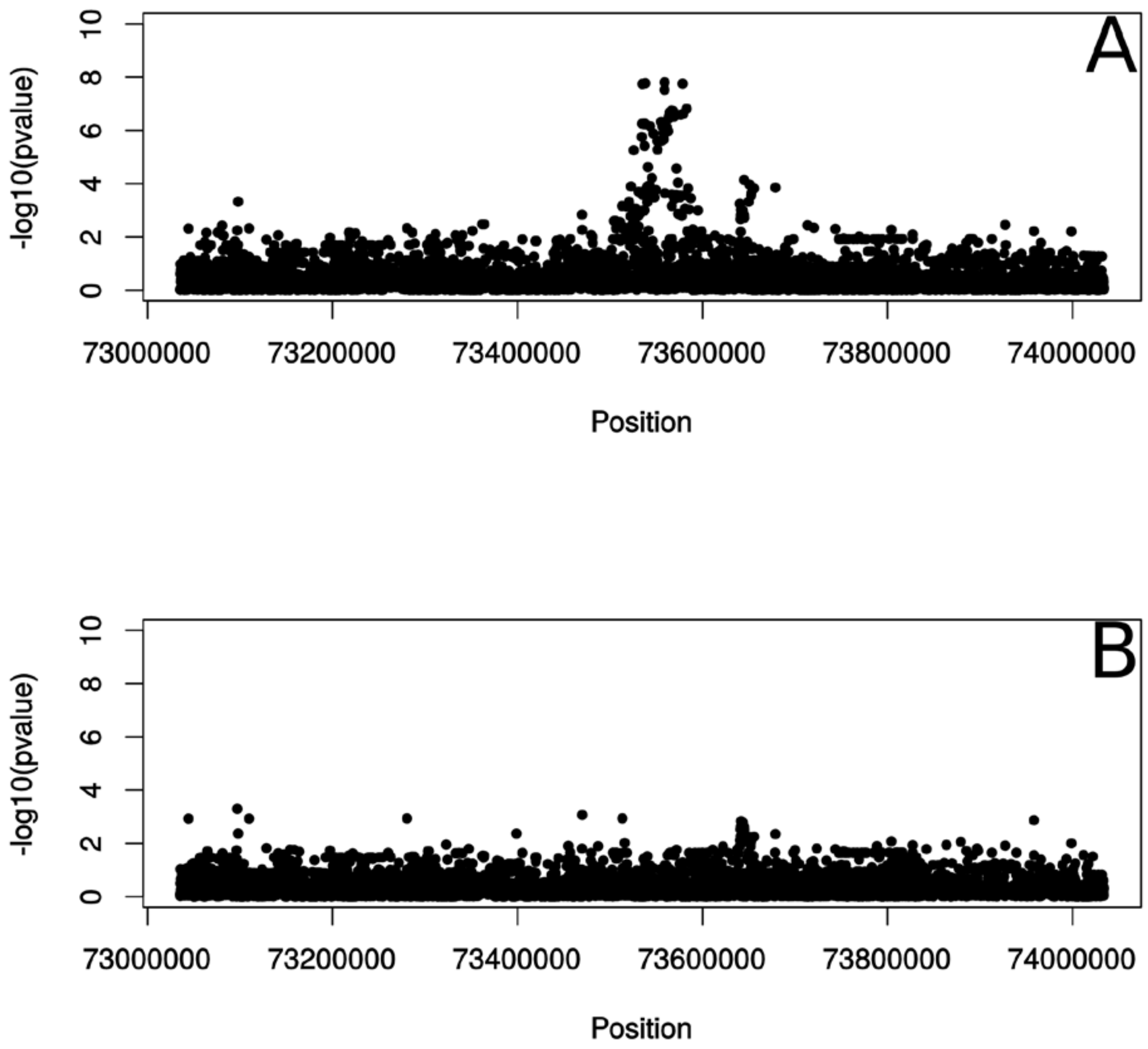

Supplementary Figure 2. Plasma PGRN association statistics on chromosome 10 locus. **(Panel A)** Manhattan plot showing the observed association statistics in the chromosome 10 locus for PGRN blood concentration using age and sex as covariates. (**Panel B**) Manhattan plot showing the observed association statistics in the chromosome 10 locus for PGRN blood concentrations using age, sex and the genotypes of SNP rs7869 as covariates.

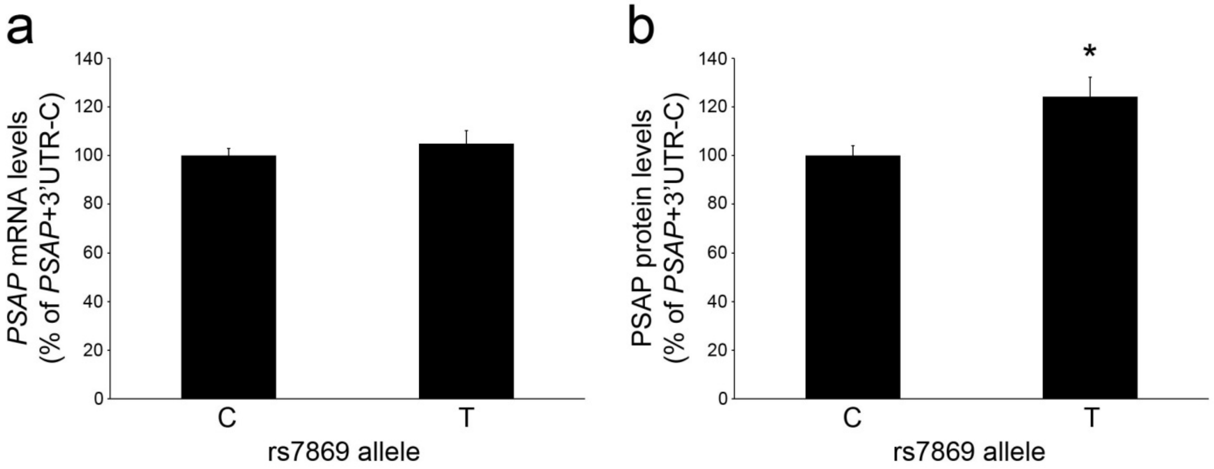

Supplementary Figure 3. SNP rs7869 regulates PSAP protein levels in transfected HeLa cells. **(a)** Quantification of PSAP mRNA levels in HeLa cells co-transfected 1 day with *EGFP*-N1 and either *PSAP*+3'UTR-C or *PSAP*+3'UTR-T plasmids (n≥11 per group). **(b)** Quantification of *GFP* mRNA levels in cells transfected as described in **(a)** (n≥11 per group). **(c)** Total PSAP protein levels were quantified (media + lysate) and normalized to GFP levels in cells transfected as described in **(a)** (n=11 per group). Graphed values represent the mean ± S.E.M. *Differs from *PSAP*+3'UTR-C, $p < 0.01$.

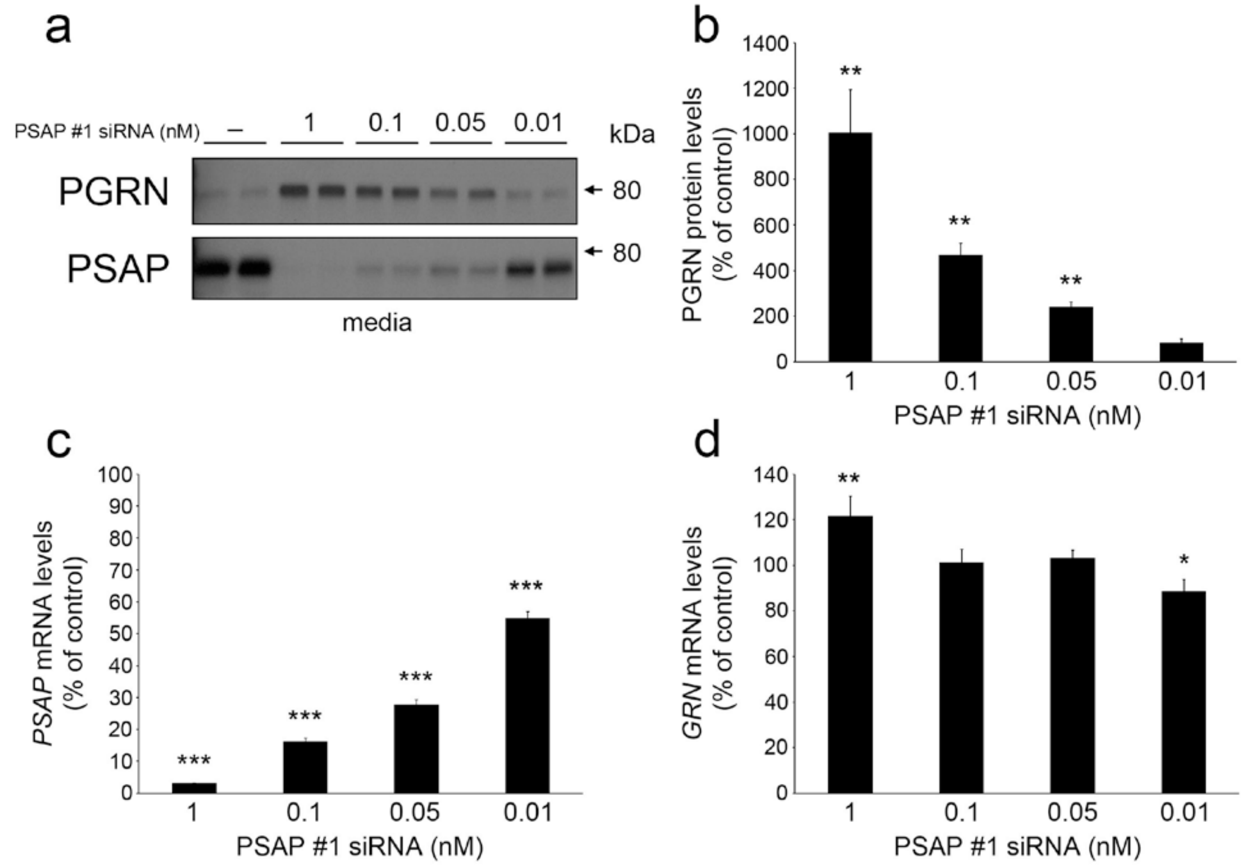

Supplementary Figure 4. PSAP-induced changes in PGRN levels is dose-dependent. (a) Western blot of conditioned media obtained from media transfected with a control siRNA (-) or with different amounts of PSAP siRNA. **(b)** Quantification of PGRN immunoreactivity in media Western blot samples ran as described in **(a)**. **(c-d)** Quantification of *PSAP* **(c)** and *GRN* **(d)** mRNA levels post transfection with a PSAP siRNA (n=6 per experiment). Graphed values represent the mean ± S.E.M. *Differs from control siRNA of the same concentration, $p < 0.05$; **$p < 0.001$; ***$p < 0.0001$.

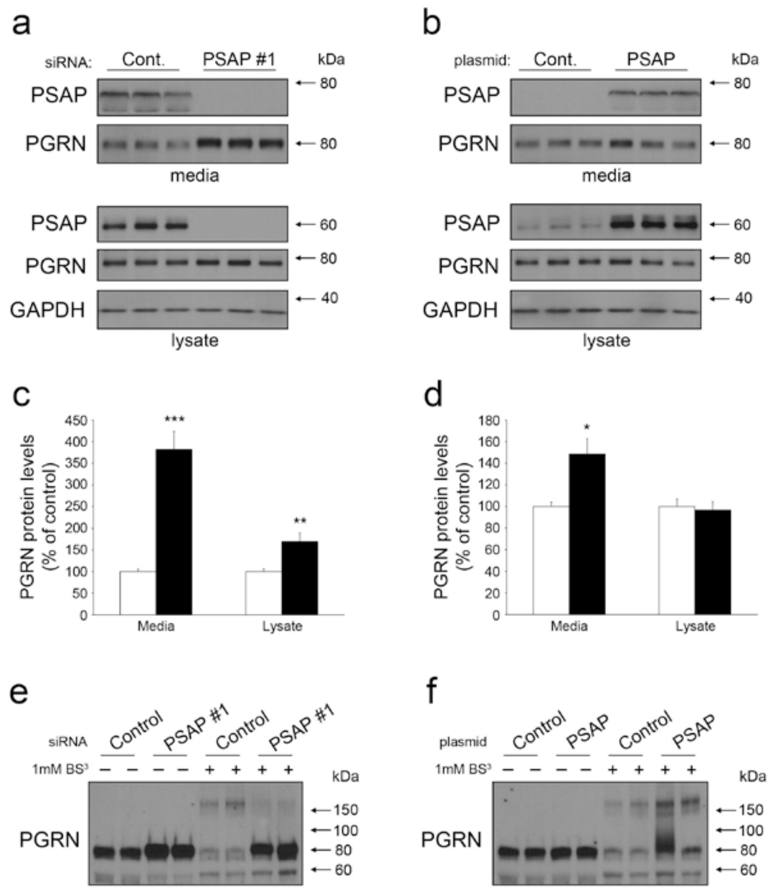

Supplementary Figure 5. PGRN levels and oligomerization are changed in human glioblastoma U251 cells. (a-b) Western blots of media and lysates obtained from U251 cells transfected with either control or PSAP siRNAs **(a)**, or with either control or PSAP plasmids **(b)**. **(c)** Quantification of PGRN immunoreactivity in media and lysates from U251 cells transfected with a control siRNA (white bars) or PSAP siRNA (black bars) (n=9 per group). **(d)** Quantification of PGRN immunoreactivity in media and lysates from U251 cells transfected with a control plasmid (white bars) or PSAP plasmid (black bars) (n≥6 per group). **(e-f)** Western blot of BS^3-crosslinked media obtained from U251 cells transfected with either control or PSAP siRNAs **(e)** or with either control or PSAP plasmids **(f)**. Graphs represent the mean ± S.E.M. *Differs from control-transfected cells of the same cell line, $p < 0.005$, $^{**}p < 0.0025$, $^{***}p < 0.0005$.

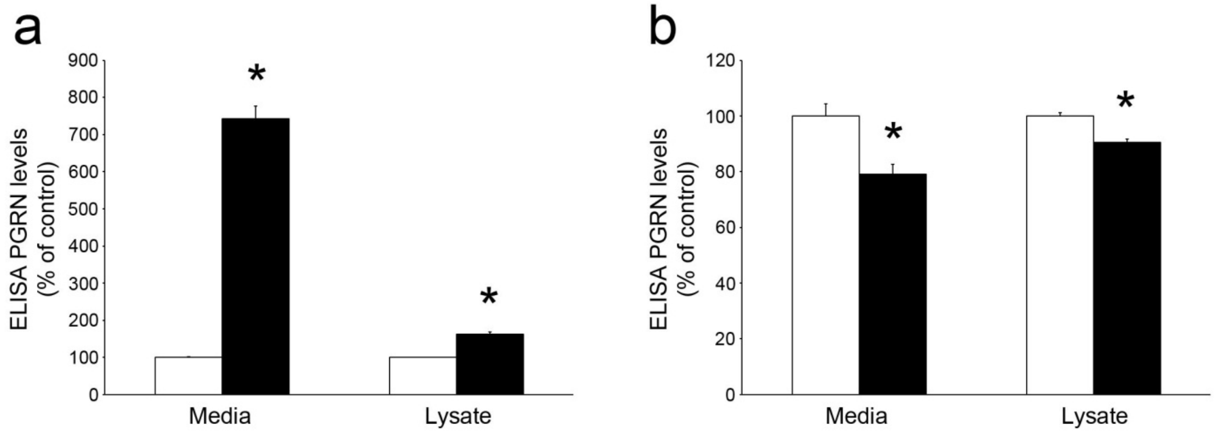

Supplementary Figure 6. PGRN measurements by the Adipogen PGRN ELISA. (a) Adipogen PGRN ELISA measurements obtained from the media or lysates of HeLa cells transfected with a control (white bars) or PSAP #1 siRNA (black bars) (n=3 per group). **(b)** Adipogen PGRN ELISA measurements obtained from the media or lysates of HeLa cells transfected with a control (white bars) or PSAP overexpression plasmid (black bars) (n=6 per group). Graphed values represent the mean ± S.E.M. *Differs from control-transfected sample, $p < 0.05$.

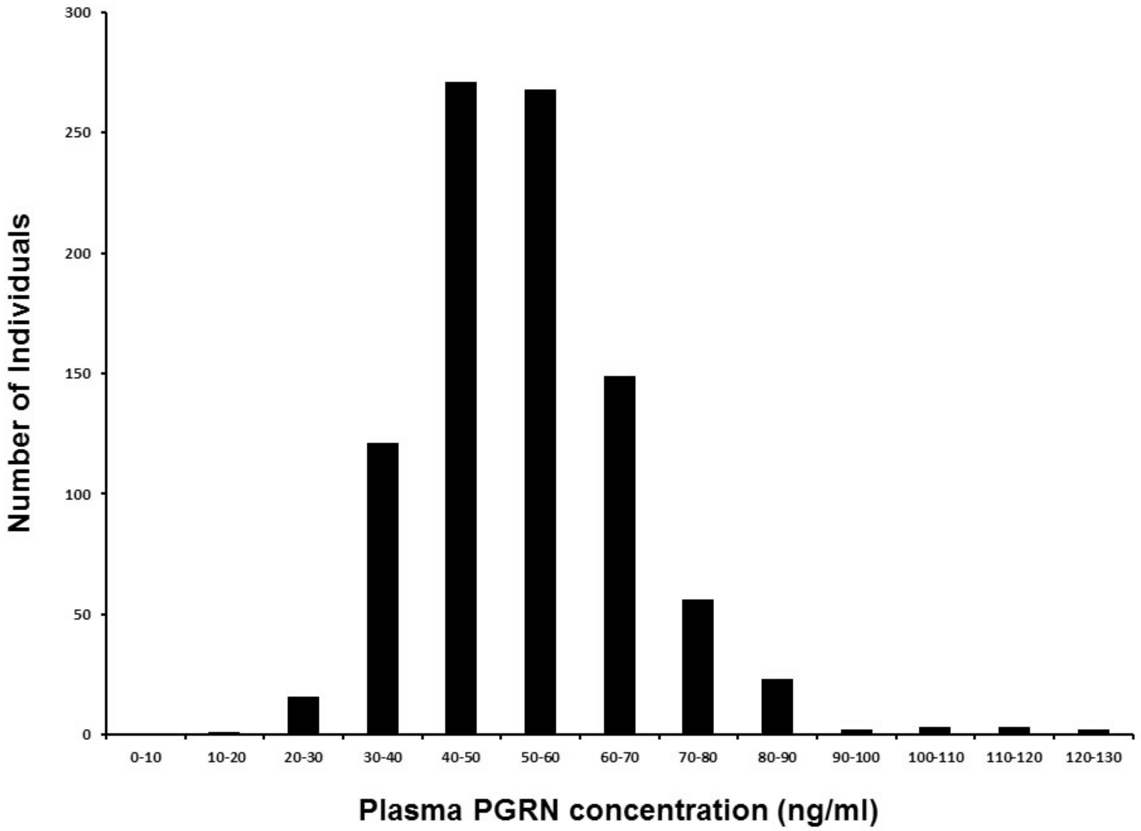

Supplementary Figure 7. Distribution of plasma PGRN levels in our initial cohort. Bar graph indicating the frequency of plasma PGRN concentrations in 10ng/ml increments using the RnD ELISA in our initial cohort (n=920).

Supplementary Figure 8. Fig. 3b full blot images

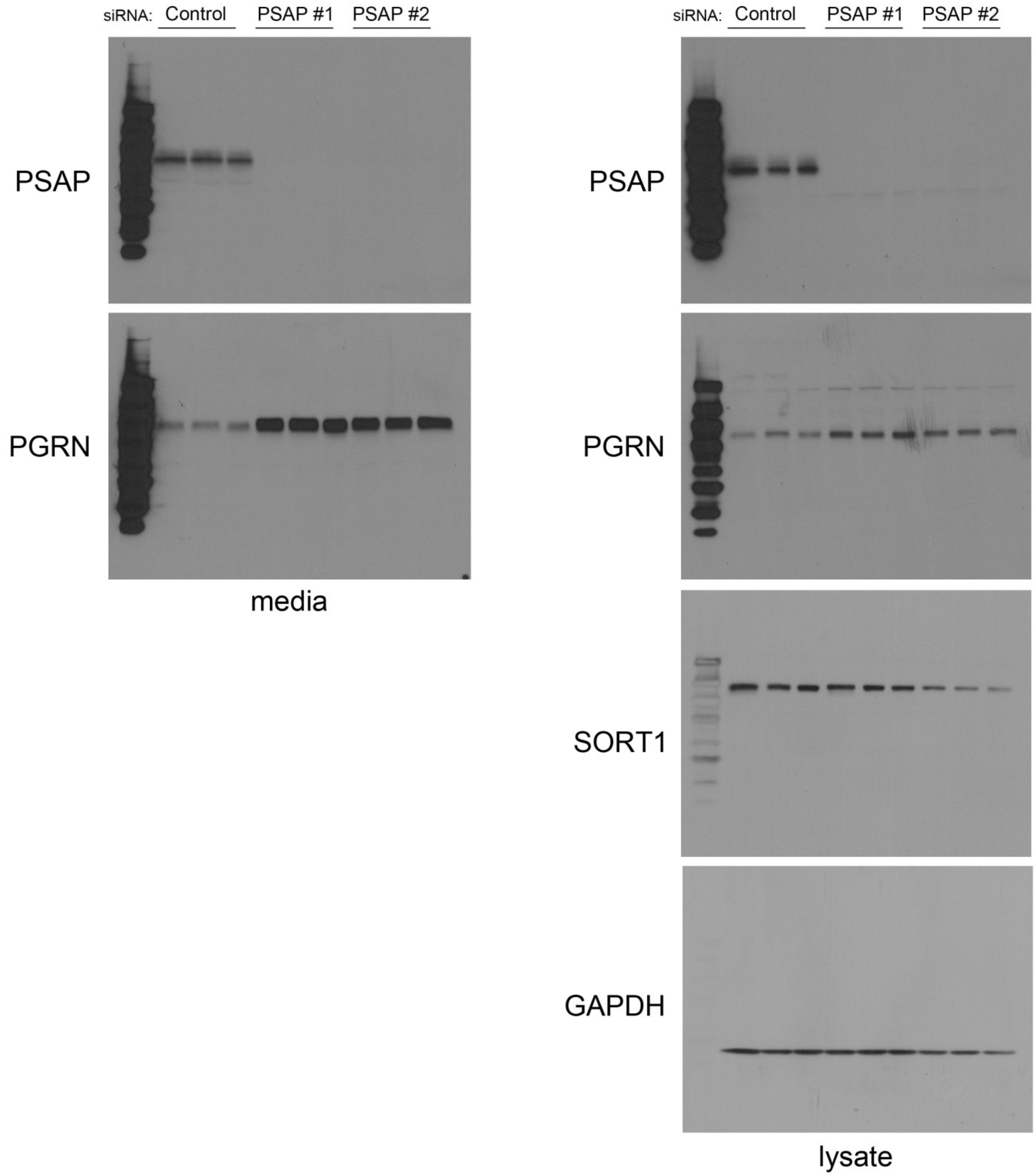

Supplementary Figure 9. Fig. 4e full blot images

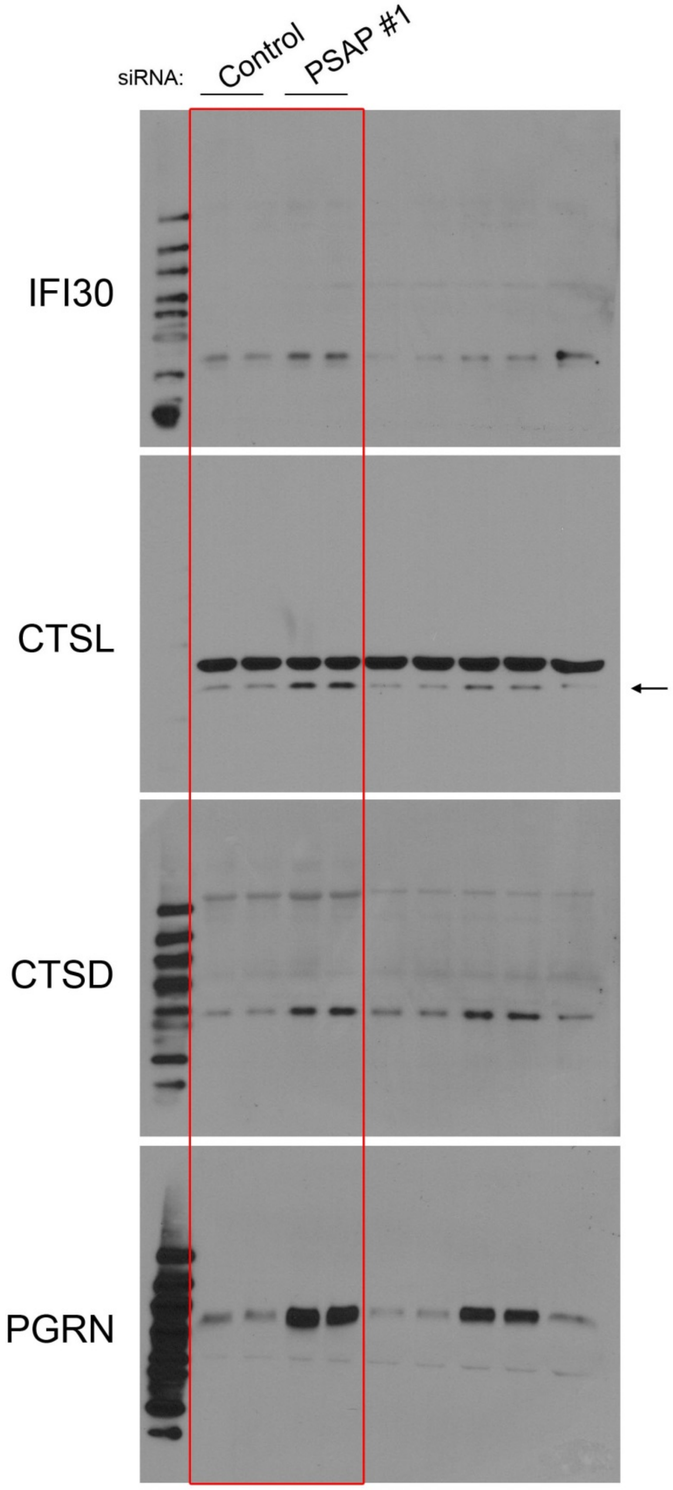

Supplementary Figure 10. Fig. 5a full blot images

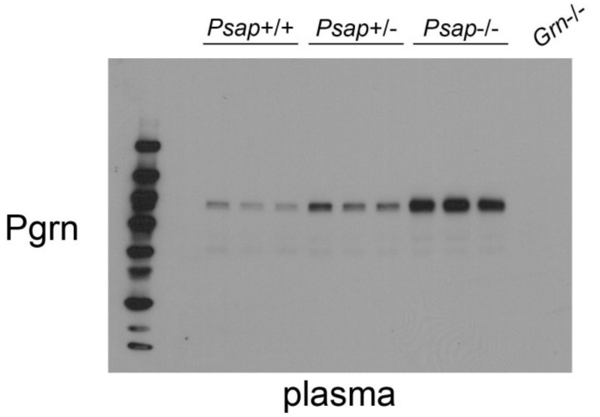

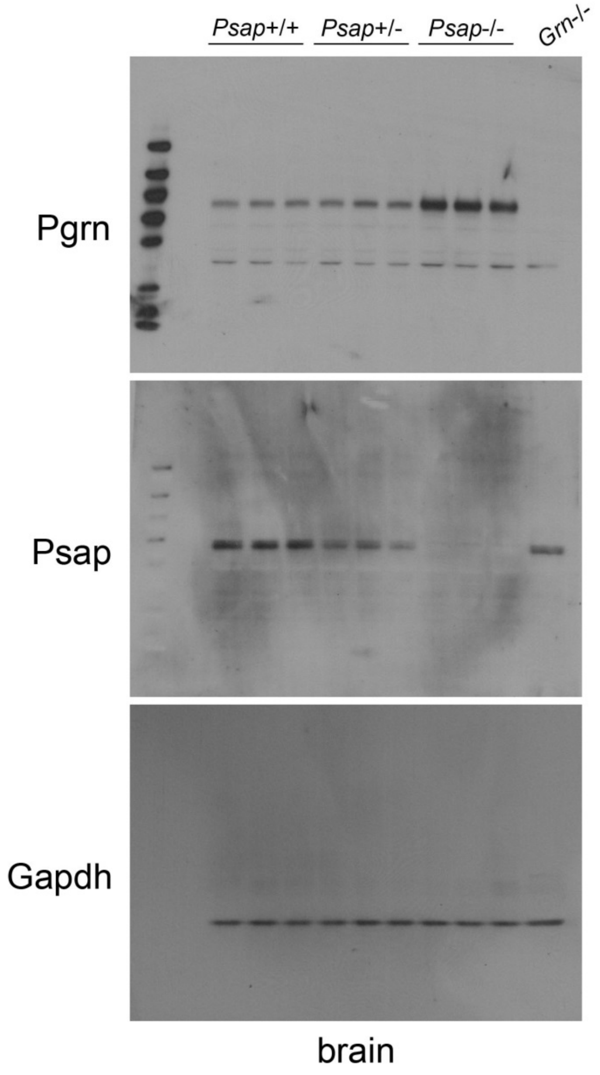

Supplementary Figure 11. Fig. 6b full blot images

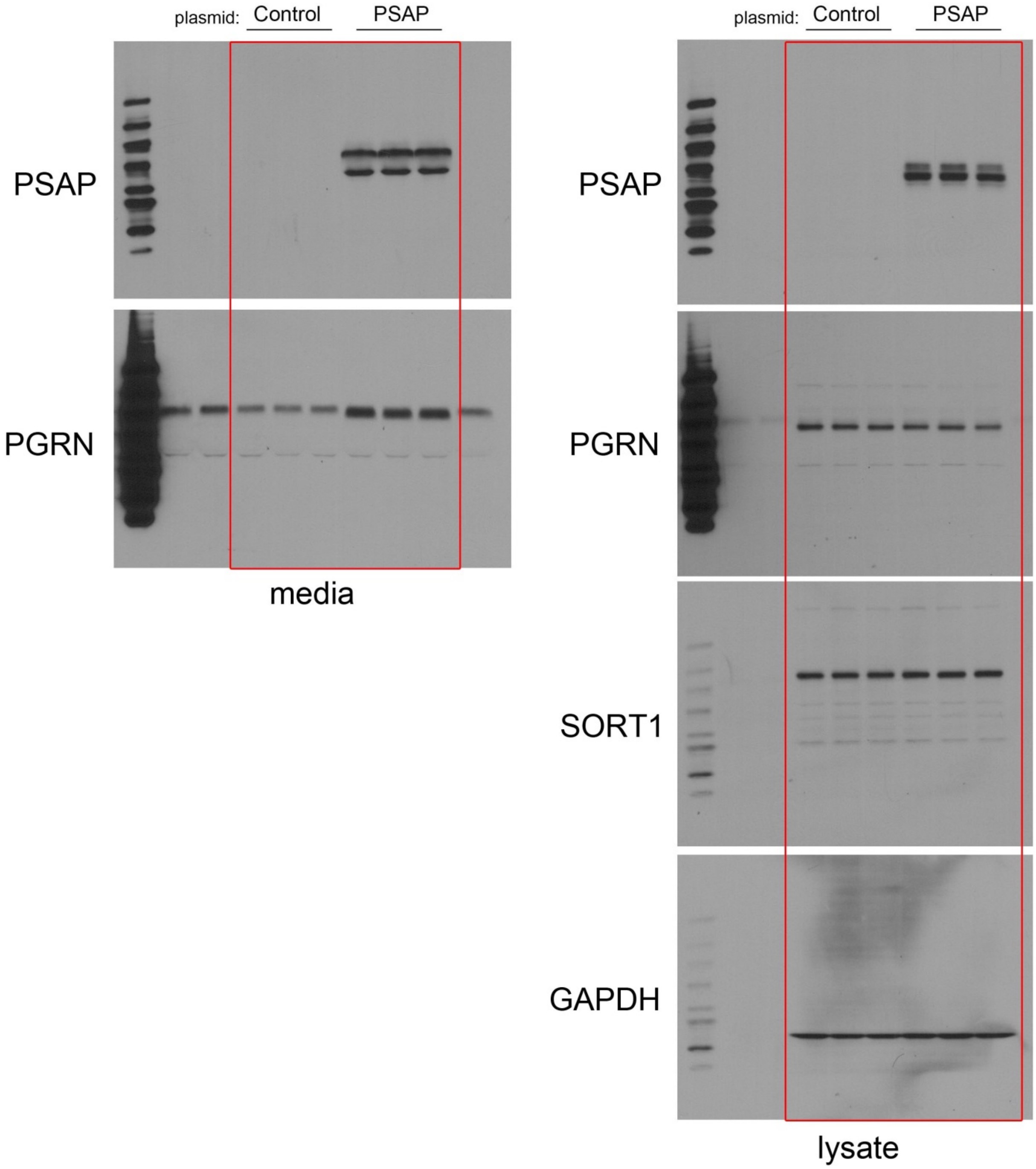

Supplementary Figure 12. Fig. 7c-f full blot images

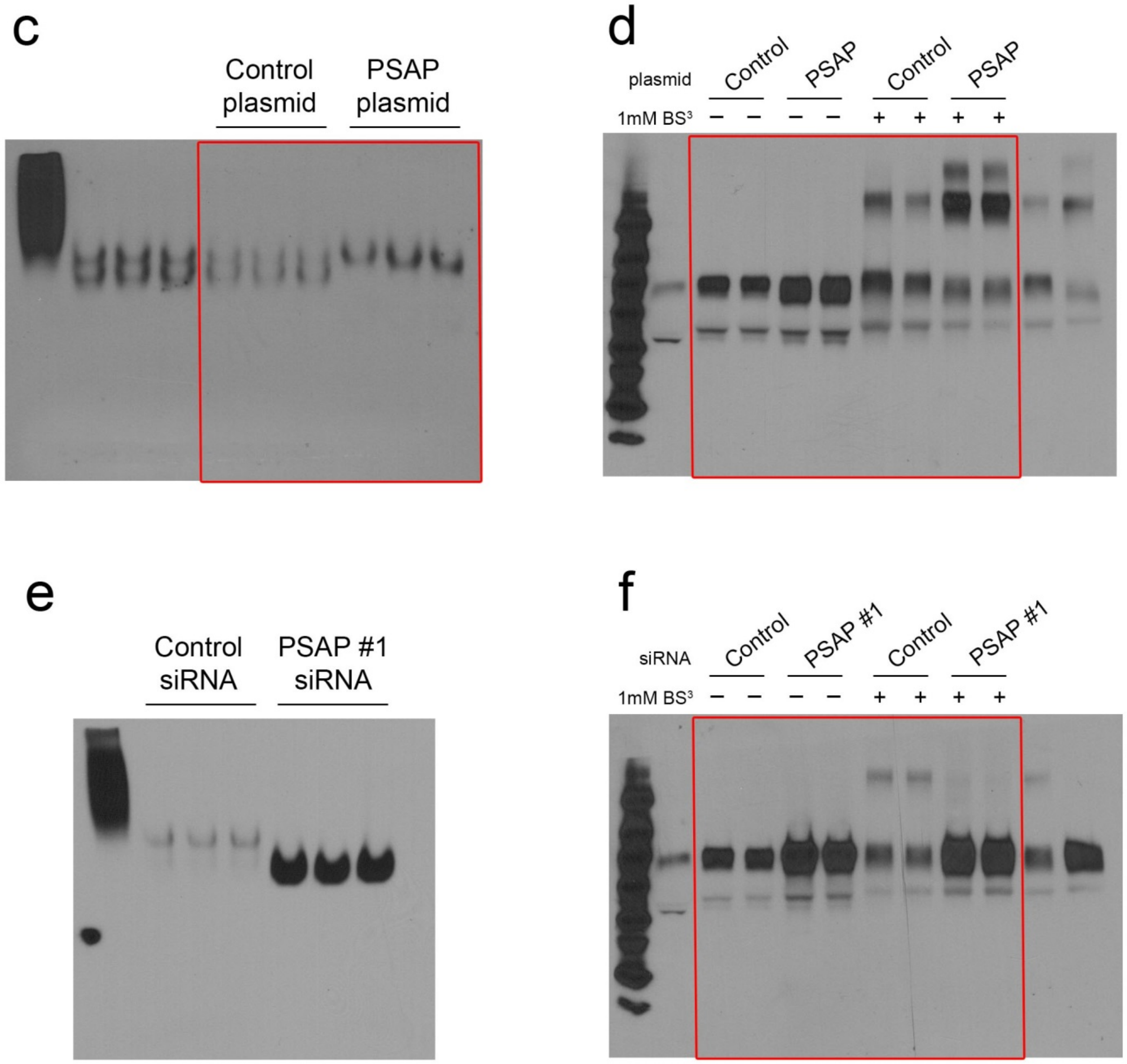

Supplementary Figure 13. Fig. 8a,b,e,f full blot images

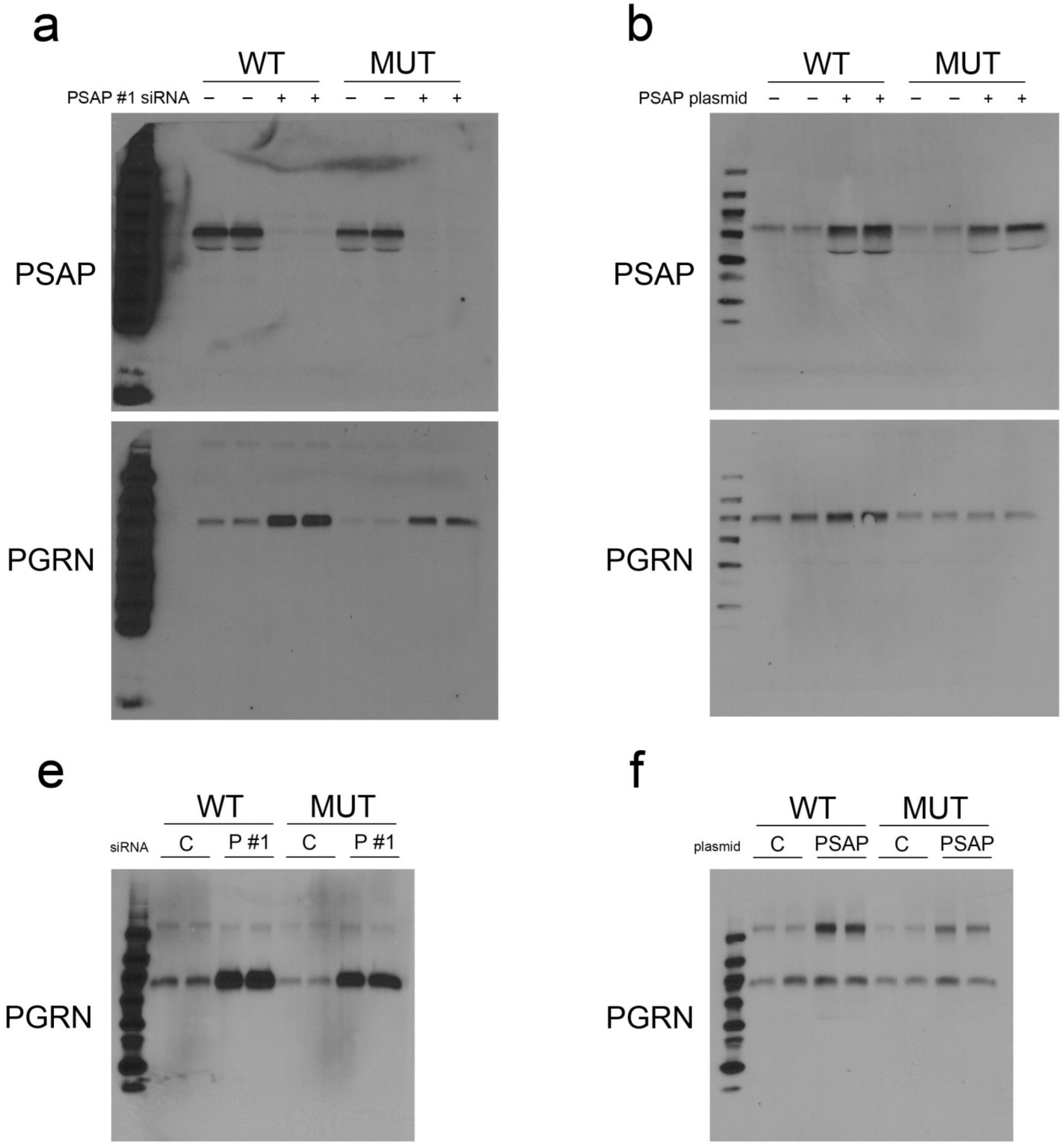

Supplementary Figure 14. Fig. 9a-c full blot images

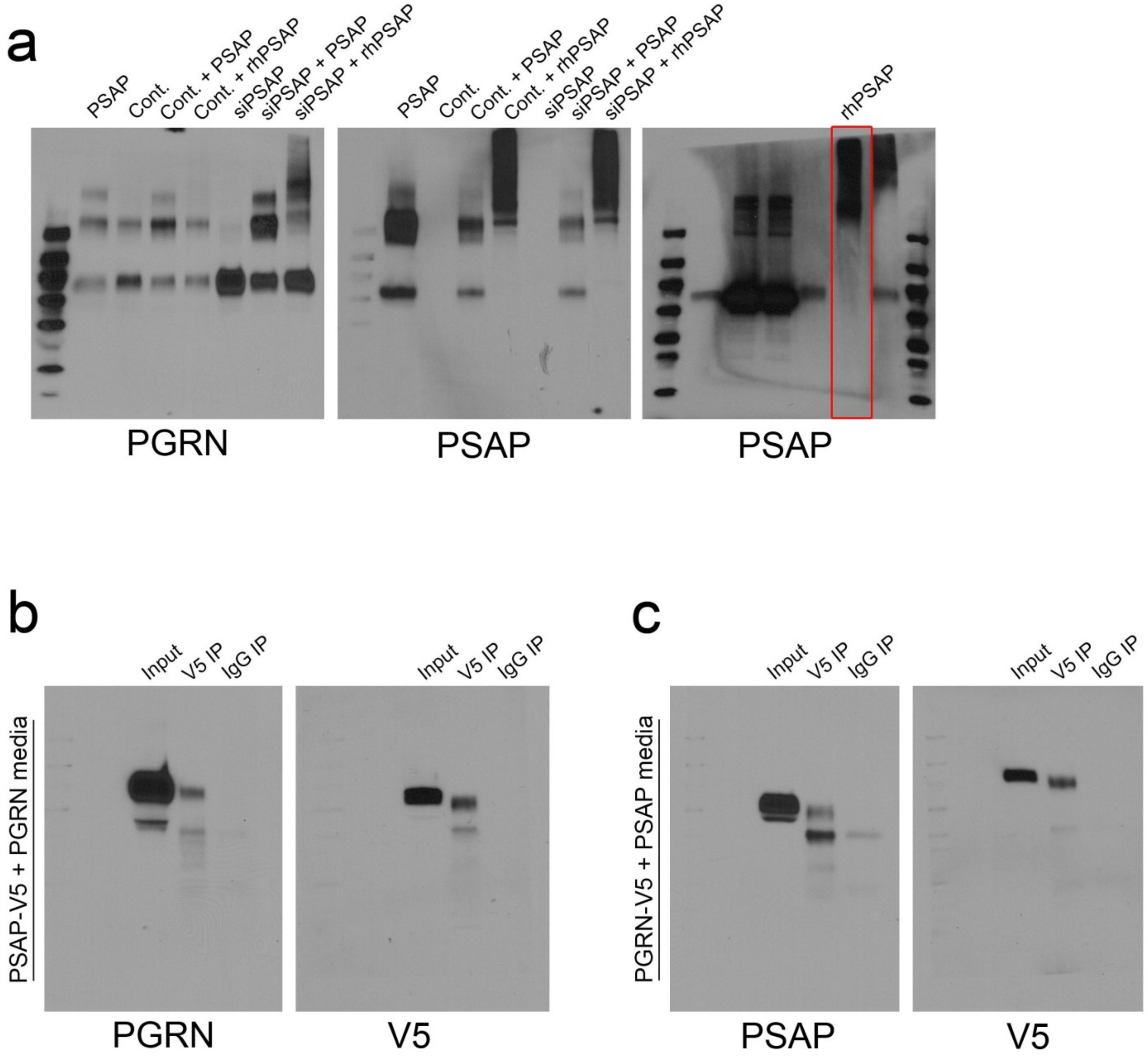

Supplementary Figure 15. Fig. 10a,b,d,e,f full blot images

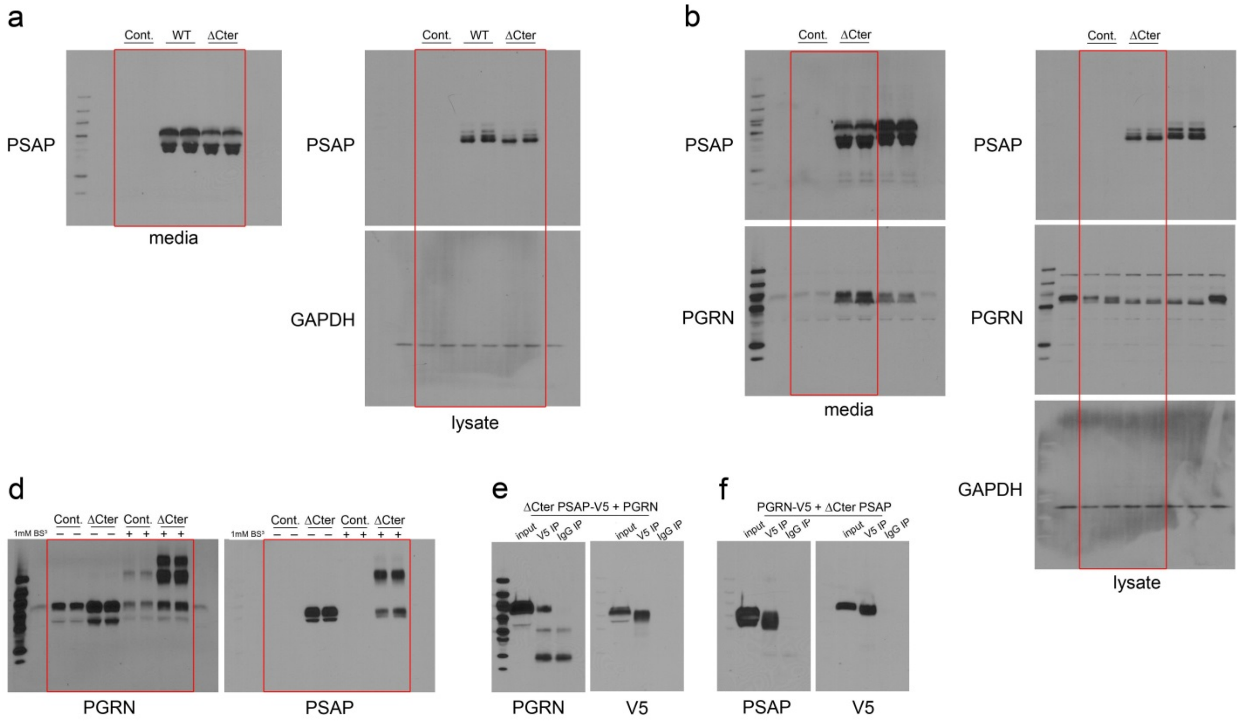

**Supplementary Table 1.** Number and type of genetic variants identified in whole genome sequence data (based on 586 individuals).		
**Variation Type**	**N (%)**	**Mean observed length of the genetic variation in bases (standard deviation)**
Deletions	858,062 (3.7%)	3.22 (4.46)
Insertions	589,767 (2.6%)	2.01 (3.09)
Single nucleotide variants	21,565,223 (93.7%)	1 (0)
Total	23,013,052 (100%)	N/A

**Supplementary Table 2.** Number of genetic variants stratified by minor allele frequency intervals in whole genome sequence data (based on 586 individuals).	
**Minor allele frequency (MAF)**	**N (%)**
MAF ≥ 10%	3,796,547 (16.5%)
5% ≤ MAF < 10%	1,079,359 (4.7%)
1% ≤ MAF < 5%	2,686,677 (11.7%)
MAF < 1%	15,450,469 (67.1%)
Total	23,013,052 (100%)

**Supplementary Table 3.** Number of genetic variants transitions and transversions in whole genome sequence data (based on 586 individuals).	
**Variation type**	**N (%)**
Transitions	14,899,828 (69.1%)
Transversions	6,665,395 (30.9%)
Total	21,565,223 (100%)

**Supplementary Table 4.** Primer Sequences	
**PSAP Sequencing Primers**	**Sequence (5'→ 3')**
PSAP-c16 Rev	GGAAGAGGGCGTACAT
PSAP-c61 For	GGACTGAAAGAATGCACCAG
PSAP-c440 Rev	TCTGCTAGGTGCTTCTGGAG
PSAP-c481 For	GAGCTGGACATGACTGAGGT
PSAP-c840 Rev	CATCTCTTTCACCTCATCAC
PSAP-c921 For	GCACGAGGTCCCAGCAAAGT
PSAP-c1184 For	GGCTGCCTGCACTGACC
PSAP-c1370 Rev	AACTGATCACACTGCTTCTGGTA
PSAP 3'UTR c991 For	GGAACTAGGAGGAGGAATAT
PSAP 3'UTR c1060 Rev	CAGACACACAAGTAGAAAAA
PSAP 3'UTR c1191 For	CTTCAGTGCCCCTTTTCTCT
PSAP3'UTR c1331 For	TCTTGGTTGAGGCCTTGTTC
PSAP 3'UTR c1470 Rev	CTTTGTTTTGCTGCTTTGTT
PSAP3'UTR c1481 For	ATGAAAGATTAGAAGCCTGG
PSAP3'UTR c1640 Rev	AGACACGGGAAAGCCAAATC
PSAP3'UTR c1870 Rev	AAAAGGTCAAAAGGAGCATC
PSAP3'UTR c1701 orF	GAATGTAACCTGCTAGCTCT
T7	TAATACGACTCACTATAGGG
BGH	TAGAAGGCACAGTCGAGG
pAAV For	GTGCTGGTTATTGTGCTGTC
pAAV Rev	CAACATAGTTAAGAATACCAGTC
**PSAP cloning Primers**	
PSAP AAV For	CCGCTCGAGGATATGTACGCCCTCTTCC
PSAP AAV V5 tag Rev	CCGAAGCTTCTACGTAGAATCGAGACCGAGGAGAGGG TTAGGGATAGGCTTACCGTTCCACACATGGCG
PSAP AAV no tag Rev	CCGAAGCTTCTAGTTCCACACATGGCG
**PSAP Mutagenesis Primers**	
PSAP dCter V5 tag Rev	CCGAAGCTTCTACGTAGAATCGAGACCGAGGAGAGGG TTAGGGATAGGCTTACCGTTCTTATGGGCCGAGGGGC AGG
PSAP dCter no tag Rev	CCGAAGCTTCTAGTTCTTATGGGCCGAGGGGCAGGCT CCAATTTTCAAGC
rs7869 mut For	CAAATAAATATGGATGGCAAGCTCCTAGGCCTCTGG
rs7869 mut Rev	CCAGAGGCCTAGGAGCTTGCCATCCATATTTATTTG
